# The Effect of ß-Glucan Prebiotic on Kidney Function, Uremic Toxins and Gut Microbiome in Stage 3 to 5 Chronic Kidney Disease (CKD) Predialysis Participants: A Randomized Controlled Trial

**DOI:** 10.3390/nu14040805

**Published:** 2022-02-14

**Authors:** Zarina Ebrahim, Sebastian Proost, Raul Yhossef Tito, Jeroen Raes, Griet Glorieux, Mohammed Rafique Moosa, Renée Blaauw

**Affiliations:** 1 Division of Human Nutrition, Department of Global Health, Stellenbosch University, Cape Town 8000, South Africa; rb@sun.ac.za; 2 Laboratory of Molecular Bacteriology, Department of Microbiology and Immunology, Rega Institute, KU Leuven, 3000 Leuven, Belgium; raulyhossef.titotadeo@kuleuven.be (R.Y.T.); jeroen.raes@kuleuven.be (J.R.); 3 Center for Microbiology, VIB, 3000 Leuven, Belgium; 4 Department of Internal Medicine and Pediatrics, Nephrology Section, Ghent University Hospital, 9000 Ghent, Belgium; griet.glorieux@ugent.be; 5 Department of Medicine, Stellenbosch University, Cape Town 8000, South Africa; rmm@sun.ac.za

**Keywords:** chronic kidney disease (CKD), gut microbiome, uremic toxins, prebiotic

## Abstract

There is growing evidence that gut dysbiosis contributes to the progression of chronic kidney disease (CKD) owing to several mechanisms, including microbiota-derived uremic toxins, diet and immune-mediated factors. The aim of this study was to investigate the effect of a ß-glucan prebiotic on kidney function, uremic toxins and the gut microbiome in stage 3 to 5 CKD participants. Fifty-nine participants were randomized to either the ß-glucan prebiotic intervention group (*n* = 30) or the control group (*n* = 29). The primary outcomes were to assess kidney function (urea, creatinine and glomerular filtration rate), plasma levels of total and free levels of uremic toxins (*p*-cresyl sulfate (*p*CS), indoxyl-sulfate (IxS), *p*-cresyl glucuronide (*p*CG) and indoxyl 3-acetic acid (IAA) and gut microbiota using 16S rRNA sequencing at baseline, week 8 and week 14. The intervention group (age 40.6 ± 11.4 y) and the control group (age 41.3 ± 12.0 y) did not differ in age or any other socio-demographic variables at baseline. There were no significant changes in kidney function over 14 weeks. There was a significant reduction in uremic toxin levels at different time points, in free IxS at 8 weeks (*p* = 0.003) and 14 weeks (*p* < 0.001), free *p*CS (*p* = 0.006) at 14 weeks and total and free *p*CG (*p* < 0.001, *p* < 0.001, respectively) and at 14 weeks. There were no differences in relative abundances of genera between groups. The redundancy analysis showed a few factors significantly affected the gut microbiome: these included triglyceride levels (*p* < 0.001), body mass index (*p* = 0.002), high- density lipoprotein (*p* < 0.001) and the prebiotic intervention (*p* = 0.002). The ß-glucan prebiotic significantly altered uremic toxin levels of intestinal origin and favorably affected the gut microbiome.

## 1. Introduction

Chronic kidney disease (CKD) prevalence is increasing globally with a prevalence of between 9 and 13% [1,2]. In sub-Saharan Africa, it is slightly higher at 14% [3], although this percentage may be underestimated owing to a lack of CKD statistics [4]. Cardiovascular disease (CVD) is the leading cause of mortality in CKD patients, despite medical treatment [5]. Reducing the progression of CKD is challenging and complex owing to the pathophysiology of the disease. The modulation of the gut microbiome has in recent years been suggested to be a therapeutic target in the management of CKD, because of its important role in kidney health [6,7,8].

The gut microbiome is home to trillions of cells of over 1000 species and is responsible for maintaining normal gut integrity and promoting immunological functions. It is involved with nutrient uptake and metabolism of nutrients, degradation of oxalates, and preventing the proliferation of harmful microorganisms [9,10]. It is considered to be an essential ‘organ’ due to its varied composition and roles in human health. In CKD, gut dysbiosis occurs because of gut wall edema, lack of fiber in the diet due to dietary restrictions, and use of antibiotics and phosphate binders, resulting in increased gut permeability [10,11]. Gut dysbiosis, in turn, contributes to uremic toxicity, inflammation and CVD [6,12,13,14]. This bidirectional relationship enhances the progression of CKD [6].

The gut-derived uremic toxins that are increased in CKD include indoxyl sulfate (IxS), *p*-cresyl sulfate (*p*CS), *p*-glucuronide (*p*CG), indole acetic acid (IAA) and trimethylamine-N-oxide (TMAO). They are associated with insulin resistance, increased oxidative stress and endothelial dysfunction [12,13,15]. The pro-inflammatory nature of these toxins results in the progression of CKD and increases mortality [14]. They originate in the colon, where aromatic amino acids are metabolized by bacteria into phenolic (*p*-cresol and phenol) and indolic compounds (indole and IAA) [16]. Phenolic compounds are produced by breakdown of tyrosine and phenylalanine, mainly by anaerobes, while indoles are produced by the bacterial breakdown of tryptophan by both aerobes and anaerobes [13].

A recent systematic review on the gut microbiome in CKD found differences in overall microbial diversity to be inconclusive between patients and healthy controls [17], but 20 microbial taxa were differentially abundant.

In general, gut microbiota can be stratified into four non-discreet community types, referred to as enterotypes [18]. The *Bacteroides* 2 enterotype, which is characterized by a large fraction of *Bacteroides* while having lower *Faecalibacterium* levels and overall microbial density, is known to be associated with inflammation [18,19]. Furthermore, *Bacteroide*s 2 has also been found to be associated with systemic inflammation linked with a high body mass index (BMI) [20] and mental health disease [21].

Pre-, pro- and synbiotics are potential therapeutic options to alter gut microbiota and reduce uremic toxin generation [22]. A systematic review reveals a low certainty of the effect of these agents owing to the varying results [22]. In addition, studies investigating the effect of prebiotics on kidney function, uremic toxins and the gut microbiome simultaneously are scarce.

Limited studies have investigated the use of prebiotics on the gut microbiome in CKD, making this an attractive therapeutic option to explore. ß-glucan, which is found in oats, seems promising to modulate the gut. Some studies in healthy individuals and those at risk of CVD showed that ß-glucan increased *Bifidobacterium* and *Lactobacillus*, reduced cholesterol levels and enhanced the production of short-chain fatty acids (SCFAs) [23,24] as well as reduced *p*CS in healthy individuals [25].

The aim of this study was to investigate the effect of ß-glucan on kidney function, uremic toxins and the gut microbiome in stage 3 to 5 CKD participants. The study hypothesis was that the prebiotic would improve kidney function, uremic toxin levels and the gut microbiome. This novel study is the first to investigate the effect of a prebiotic on all of these outcomes.

## 2. Materials and Methods

This randomized control trial (RCT) was a single-center, single-blinded study investigating the effects of a ß-glucan prebiotic on kidney function, plasma levels of uremic toxins and the gut microbiome. Participants were randomized to either receive a daily prebiotic supplement together with CKD dietary advice (intervention group) or to remain on the diet only (control group). Ethics approval was obtained from the Health Research Ethics Committee of Stellenbosch University (S18/03/064), and the study adhered to the Declaration of Helsinki principles. The protocol submitted for ethics approval was adhered to during the study. Informed consent was obtained from participants before they enrolled in the study. The study was registered with the Pan African Trial Registry (PACTR202002892187265).

### 2.1. Participants

**Figure 1:**
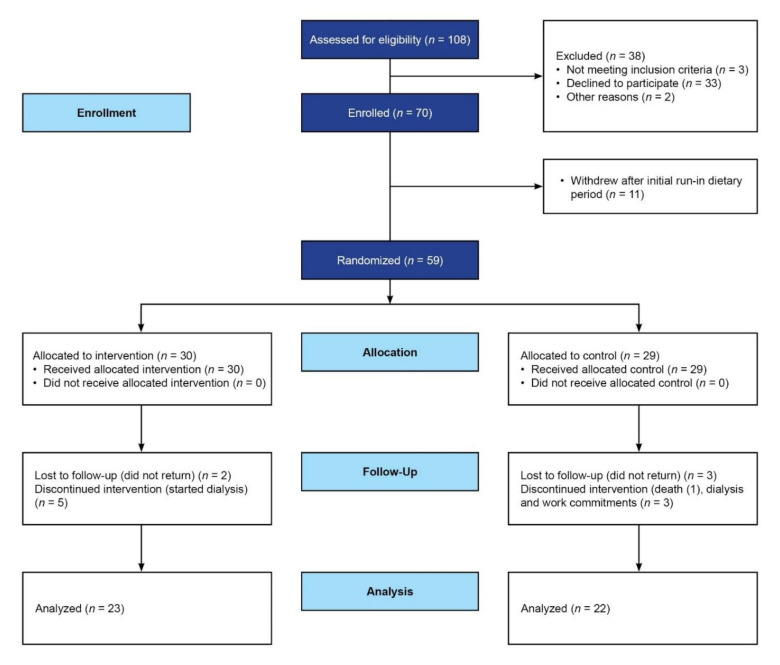
CONSORT flow diagram on the effects of a ß-glucan prebiotic on kidney function, plasma levels of uremic toxins and the gut microbiome in predialysis participants with CKD stage 3 to 5.

Participants (over 18 years) attending a predialysis clinic in Cape Town, South Africa, with CKD stage 3 to 5 (classified by a GFR< 60 mL/min per 1.73 m^2^) were enrolled. Only participants over 18 years old were recruited. Participants were excluded if they met the following criteria: taking antibiotics, prebiotics or probiotic supplements currently or in the past four weeks, participants with inflammatory bowel disease, bowel malignancy, previous colorectal surgery (or any other serious bowel disorder), pregnancy, diabetes mellitus, coeliac disease, human immunodeficiency virus (HIV) disease, malignant hypertension, crescentic glomerular nephritis, participants on immunosuppressant medications, and those expected to start immediate dialysis. Figure 1 shows the CONSORT flow diagram for progress of participants through the trial. There were 108 participants eligible for inclusion, of whom 70 participants were enrolled for the pre-randomization period. Eleven were lost to follow-up at the baseline, with only 59 participants being randomized.

### 2.2. Design

**Figure 2:**
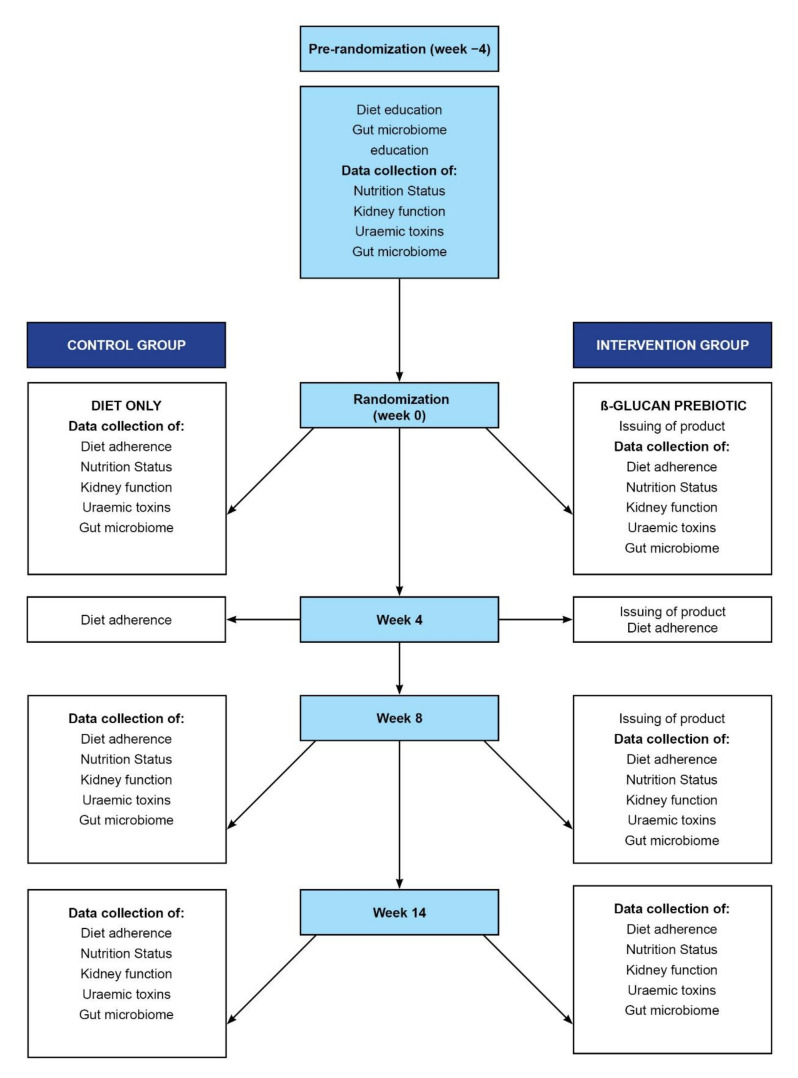
Schematic representation of the study flow; Nutrition status includes anthropometry, clinical, dietary assessment, other nutrition-related biochemical tests.

As depicted in Figure 2, all participants had a four-week run-in period on diet only before being randomized to the prebiotic supplement or the diet control group using a simple computer-generated randomized list with an equal allocation ratio. Sequentially numbered sealed opaque envelopes were used to assign group allocation. The principal investigator (PI) was blinded to the treatment. Participants had follow-ups at week 4 (only for issuing product and checking adherence to the diet), week 8 and week 14. They were therefore on the intervention or control for 14 weeks. Nutritional assessments (including anthropometry, biochemical, clinical and dietary intake and adherence scores), blood samples for quantification of uremic toxins and stool samples for characterization of the gut microbiome were obtained at each of the time points, except for week 4. Education of the gut microbiome stool sample collection and completion of the Bristol Stool Scores (BSS) was done at pre-randomization.

### 2.3. Sample Size

Sample size was calculated to estimate a change from baseline to the end of the intervention of 12% in urea, 5% in creatinine and an increase in Lactobacillus of 20% using a two-sample t-test using the Power Analysis and Sample Size software (PASS program), based on a previous study [26]. A 90% power was used. The estimated numbers to detect the change were 16 for urea, 15 for creatinine, and 23 for *Lactobacillus* in each arm of the trial. Therefore, 23 were the minimum required in each group. To compensate for possible withdrawals during the study, 70 participants were enrolled.

### 2.4. Anthropometry and Biochemistry Methods

The following measurements were performed by the researchers at all three time points: weight, height, waist circumference, and mid-upper arm circumference using standard measures [27]. BMI was calculated, and waist and BMI were interpreted according to WHO standards [28]. Blood was collected by the clinic nurse and sent to the National Health Service laboratory for biochemical analysis. Biochemical tests included kidney function tests, lipid function tests, electrolytes and C-reactive protein (CRP). Blood pressure measurements were performed by the clinic nurse. Participants were asked about gastrointestinal symptoms according to a Likert scale of ‘never’, ‘mild’, ‘moderate’ or ‘severe’ for the following: nausea, vomiting, flatulence, anorexia, constipation and diarrhea.

### 2.5. Dietary Intake, Education and Prebiotic Product

The researchers gave in-person dietary advice based on evidence-based CKD guidelines to all patients. The detail of the dietary education has already been described [29]. In short, this entailed education on a simplified diet based on natural, healthy food with the avoidance of take-away, salt and processed foods rich in additives. A protein restriction of 0.8 g/kg was advised. The participants were advised on the diet four weeks before they were randomized to ensure that changes that occurred in the study were not due to dietary changes. The diet was adhered to during the study.

Participants in the IG were advised to use 13.5 g of ß-glucan prebiotic fiber supplement (GlucaChol-22^®^; manufacturer—At Life Products, Bryanston, South Africa) containing 6 g of fiber of which 3 g is ß-glucan per serving) daily. Dietary tips were given to use the prebiotic supplement in various ways, such as adding it to smoothies, cereals or yoghurts. Participants were given sufficient prebiotic supplement until their next appointment. The PI was responsible for the measurements of participants and assessment of dietary intake. To ensure that the PI remained blinded to the study intervention (the prebiotic supplement), the research assistant was responsible for the issuing of the supplement. Product adherence was measured by the return of empty containers.

Dietary intake was measured at all three time points using an interview-administered 160-item CKD adapted quantified food-frequency questionnaire (FFQ). Portions were calculated to a daily intake and analyzed using the SA foods database [30]. An adapted dietary adherence score sheet was used to measure dietary adherence. There were 12 questions on dietary changes from various food groups with a set of criteria which were selected based on information advised in the infographic and the dietary advice given. The detail of this score sheet and the FFQ have been described [29]. Participants scored a point if they adhered to the criteria, with a maximum of 12 points. An adherence of 10–12 (85–100%) was classified as excellent adherence, while 8–9 (67–75%) was good adherence, 6–7 (50–58%) was average adherence and <5 (<50%) was considered poor adherence.

### 2.6. Uremic Toxin Quantification

For the quantification of uremic toxins (UTs), venous blood was collected at all time points (except week 4) in K-EDTA tubes (9 mL). Blood was immediately centrifuged at 2100× *g* for 10 min at 4 °C. Plasma was aliquoted on ice at 500 µL in sterile tubes, then stored at −80 °C before it was sent on dry ice to the Nephrology laboratory of the Ghent University Hospital in Belgium for batch analysis. Total and free concentrations of *p*CS, IxS, *p*CG and IAA were determined by liquid chromatography and fluorescence detection as previously described [31]. In brief, for quantification of the total toxin concentrations, plasma samples were deproteinized by heat, centrifuged and filtered through an Amicon^®^ Ultra 0.5 µL (Merck Millipore Ltd. Carrigtwohill, Ireland) (molecular weight cut-off 30 kDa filter). For the free fraction, the untreated plasma was first filtered through the Amicon filter. The ultrafiltrate was transferred into an autosampler vial, and fluorescein was added as an internal standard. Analysis was performed by ultra-performance liquid chromatography with an Agilent 1290 Infinity device (Agilent, Santa Clara, United States of America). IxS (λex: 280 nm, λem: 376 nm), *p*CS and *p*CG (λex: 264 nm λem: 290 nm), IAA (λex: 280 nm, λem: 350 nm), and fluorescein (λex: 443 nm, λem: 512 nm) was detected by an Agilent G1316C fluorescence detector.

### 2.7. Stool-Sample Collection

All participants were given adequate instructions and stool-sample collection kits together with ice packs and a cooler bag. An information leaflet was also provided. They were asked to collect the sample the day before the study appointment and asked to freeze the sample immediately after collection. After their appointment, the samples were immediately frozen at −80 °C. Participants were also asked to complete the BSS which explains the type and consistency of the stool, ranging from 1 to 7 (from very hard to very loose), the time of the sample collection, and the time of their last stool. The samples were sent on dry ice to VIB laboratories at KU Leuven, Belgium, for further analysis.

### 2.8. Analysis of Gut Microbiome

To analyze microbiota taxonomic composition, fecal DNA extraction, library preparation and 16S ribosomal RNA (rRNA) gene sequencing were performed as described by Tito et al. [32] using the MiSeq platform at VIB laboratories in Leuven, Belgium.

The gut microbiome results were analyzed using the 16SRNA amplicon method by amplifying the V4 region of the 16S rRNA gene with the 515F and 606R primer (GTGYCAGCMGCCGCGGTAA and GGACTACNVGGGTWTCTAAT, respectively) to produce dual barcoded libraries. This modification was done to contain a barcode sequence between each primer and the Illumina adaptor sequence [33]. Before the sequencing, size selection was performed using Agencourt AMPure (Beckman Coulter, Indianapolis, United States of America) to remove fragments below 200. The Illumina MiSeq platform (Illumina, San Diego, United States of America) (MiSeq Reagent Kit v2, 500 cycles, 15.38% PhiX, 2 × 250 PE) was used. Fastq sequences were further analyzed per sample using the DADA2 pipeline [34]. This was done after de-multiplexing with sdm, while no mismatching was allowed as part of the LotuS pipeline [35]. After inspecting for quality, sequences were trimmed to remove the primers and the first 10 bases of the primer, which resulted in only 200 bases for R1 files and 130 for R2 files. After removing chimeras and merging sequences, taxonomy was assigned using the formatted RDP training set, ‘rdp_train_set_16’.

### 2.9. Statistical Analysis

Baseline data were described using means and standard deviation for normally distributed continuous variables and medians, and interquartile ranges for non-normally distributed variables. Frequencies were used for categorical data. Data were checked for normality using Kolmogorov tests, histograms and skewness values. To control for proper randomization, baseline comparisons between the intervention and the control groups were done using t-tests for normally distributed data and Mann–Whitney tests for non-normal data. Categorical data were compared using chi-square tests.

Generalized estimating equation (GEE) models were fitted to identify time effects and differences between the intervention and control group (treatment effects) in terms of kidney function, uremic toxin levels and gut microbiome. In all models, we included the time point (baseline, week 8 and week 14), the treatment group (treatment and control) variables and their interaction as predictors.

A log link with a gamma distribution for the residual was chosen to model severely non-normal variables, including biochemistry, uremic toxin levels and dietary data. An identity link with the normal distribution of residuals was used to model anthropometric data. The models were fitted by using a Huber–White robust estimator with an unstructured correlation matrix.

All analyses were conducted using IBM^®^SPSS^®^ version 27 (10 November 2021). A 5% level of significance was used to reject the null hypothesis in statistical tests (two sides). A *p*-value of <0.05 was considered significant.

### 2.10. Gut Microbiome Statistical Analysis

Statistical analyses were performed using R Statistical Software version 3.6.1 (http://www.r-project.org/, accessed on 15 September 2021).

Prior to analysis, 16S reads were rarefied to an even depth of 10,000 reads per sample; genera with low prevalence (present in less than 20% of the samples) were excluded from further tests. Alpha diversity, using the Shannon Diversity Index, was calculated using an in-house script. The Principal Coordinate Analysis and other analyses based on the Bray–Curtis distances were done using Scikit-learn (v0.24.1) and Scikit-bio (v0.5.5) using Python (v3.8.2) [36,37]. Comparisons between groups were made using the package statannot, which applies a pairwise Kruskal–Wallis test with Bonferroni correction for multiple testing. Genus-level, microbiome profiles variation explained by various features in the metadata was performed using univariate or multivariate stepwise distance-based redundancy analysis as implemented in R (v3.6.1) using the rda function from the vegan (v2.5-6) package [38]. Group-wise comparisons at genus level were performed using ALDEx2 (v1.18.0, ran using R v3.6.1), with mc.samples = 256, leaving other parameters at their default values [39]. The function T-test was used with default parameters (either in paired or unpaired mode, depending on the comparison). Correlations between genera and metadata were established using FlashWeave (v0.18 ran with Julia 1.6.1), using default parameters [40].

Samples’ community types, hereafter referred to as enterotypes, were obtained by combining 16S rRNA gene data from this cohort with data from the Flemish Gut Flora Project (FGFP) [35] and applying an approach based on Dirichlet Multinomial Mixtures (DMM) [36].

## 3. Results

### 3.1. Baseline Data

Fifty-nine participants were enrolled in the trial from August 2018 to December 2019; 30 were randomized to the intervention group and 29 to the control group (Figure 1). Baseline characteristics are described in Table 1. Participants had a mean age of 41.0 ± 11.9 years and were predominantly female. The main cause of kidney failure as documented in the medical files was hypertension. Half of the participants were unemployed, with most participants having a monthly income of less than US $126.

The overall BMI was 28.3 ± 6.6 kg/m^2^, with a high prevalence of overweight and obesity in 67.7% of participants. Biochemistry and the plasma levels of uremic toxins are shown in Table 2 and are reflective of CKD.

The baseline protein intake percentage was 15% of energy intake, which was 0.7 g per kg of ideal body weight for the group at baseline, carbohydrate intake was 56% and fat intake 33% of energy intake, of which saturated fat intake was 8.6%. Fiber intake was 17.6 g (14.1, 21.3), while total sugar intake was 61.5 ± 23 g. Mineral intake is shown in Table 1.

There were no significant differences between the intervention and the control group in baseline characteristics except for slightly higher high-density (HDL) levels in the intervention group.

### 3.2. Post-Intervention Data

Forty-five participants completed both arms of the trial in full. (Figure 1).

### 3.3. Kidney Function and Biochemistry

There were no significant changes in serum urea and creatinine concentrations or glomerular filtration rate over 14 weeks (Table 3). There was a significant reduction in low-density lipoprotein (LDL) cholesterol in the intervention group (Table 4), with a significant treatment effect. At week 8, the decrease in the intervention group was 0.88 times the decrease in the control group, but at week 14 the difference disappeared. Total cholesterol, HDL cholesterol and triglycerides (TG) did not change (Table 4). All the other biochemical values, including potassium, phosphate, sodium, and CRP, remained unchanged throughout the study (Supplementary Table S1). CRP was raised (>3 mg/L) in 64% of participants.

### 3.4. Plasma Levels of Colon-Derived Uremic Toxins

There was a significant decrease of free IxS over time in the intervention group, with a significant treatment effect (Table 5). The decrease in the intervention group was 0.46 times the decrease in the control group during week 8 (*p* = 0.003), and 0.35 times during week 14 (*p* < 0.001). Supplementary Figures S1–S3 graphically show the mean change over time in uremic toxins.

There was a significant intervention effect on free *p*CS; the decrease in the intervention groups was 0.48 times the decrease in the control group at week 14 (*p* = 0.006).

There was a significant decrease in total *p*CG and free *p*CG at week 14, with a significant intervention effect. For total *p*CG, the decrease in the intervention group was 0.14 times the decrease than in the control group (*p* < 0.001) at week 14, and for free *p*CG, the decrease in the intervention group was 0.13 times the decrease than in the control group (*p* < 0.001) at week 14. Free IAA was 0.56 times the decrease in the intervention vs. the control group at week 14, with it being very close to statistical significance (*p* = 0.051).

### 3.5. Anthropometry, Dietary Changes and Adherence to the Diet

There were no significant anthropometrical, and dietary changes from baseline to the end of the study (Supplementary data—Tables S2 and S3).

Diet adherence was excellent (above 85% throughout) and improved as the study progressed (Supplementary data—Table S4).

Compliance with the product as measured by the return of empty containers was good, with an 89.6% compliance at week 4, a 90.4% compliance at week 8, and an 82.7% compliance at the end of the study. Some participants did not return their containers, although they reported that they were using the prebiotic. Nearly all participants (>90%) did not experience gastrointestinal symptoms such as nausea, vomiting, flatulence, anorexia, constipation and diarrhea at baseline, and this did not change over time between the two groups.

### 3.6. Gut Microbiome

**Figure 3:**
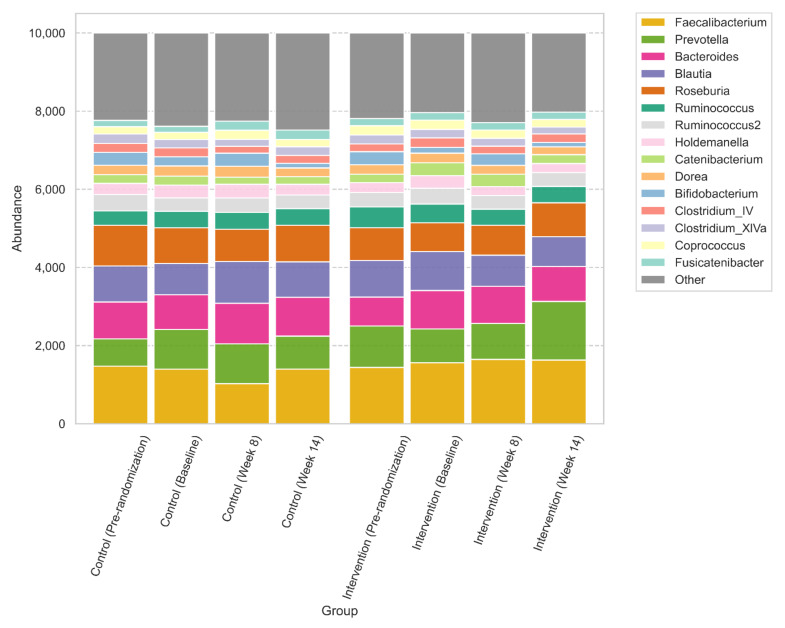
Relative abundances at the genera level.

The most abundant genera were *Faecalibacterium*, *Prevotella*, *Bacteroides*, *Blautia* and *Roseburia* as shown in Figure 3. Although there was a trend to increase in *Prevotella* and a trend to reduction in *Bacteroides* and *Blautia* in the intervention group, this was not significant after correcting for multiple testing using ALDEx2. (Supplementary data—Table S5).

Gut microbiota were characterized by a high relative abundance of *Prevotella* and *Bacteroides,* as shown in Figure 3. This was further confirmed by enterotyping using the Flemish Gut Flora Project as a background (Supplemental Figure S4), where a large subset of participants were assigned to the Prevotella enterotype [41]. There were no significant differences in relative abundances between groups using ALDEx2 (Supplementary data—Table S5).

**Figure 4:**
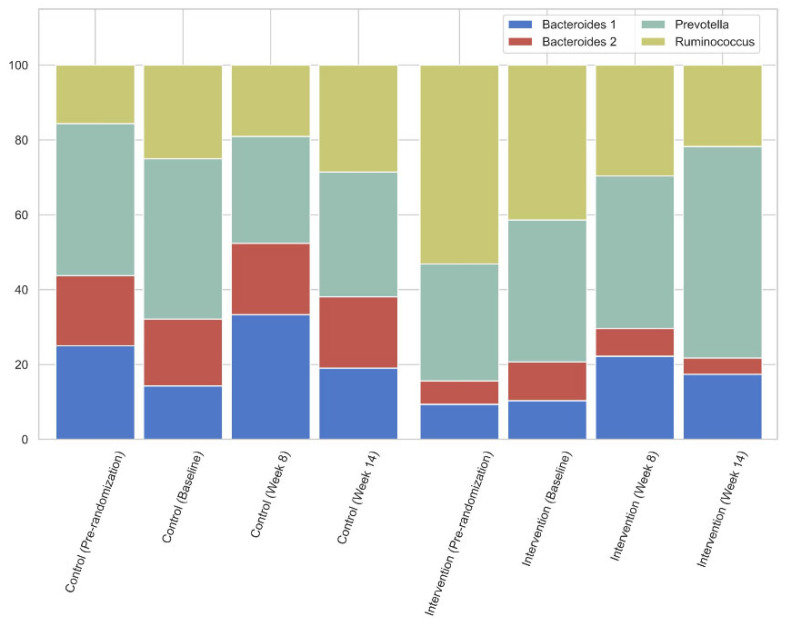
Enterotype percentages of the intervention and control group over time.

When comparing the intervention and the control group, as shown in Figure 4, there was a slight downward trend in Prevotella prevalence while increasing in the treatment group.

**Figure 5:**
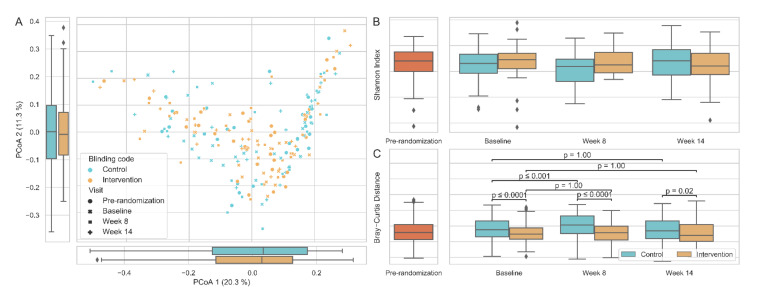
Alpha and beta diversity comparisons by groups. (**A**) Principal coordinate analysis (PCoA2) of inter-individual differences (beta diversity) by Bray–Curtis dissimilarity. (**B**) Alpha diversity according to the Shannon index. (**C**) Within-group inter-individual Bray–Curtis distance.

Figure 5A shows the principal coordinate analysis of inter-individual differences (beta diversity) by Bray–Curtis dissimilarity. The inter-individual Bray–Curtis distance was significantly higher in the control group than the intervention group at baseline (*p* < 0.0001) and remained higher throughout the total study period. Furthermore, a significant difference was observed between the baseline and week 8 in the control group (*p* < 0.001) (Figure 5B). There were no differences in alpha diversity between the intervention and control group (Shannon index) at randomization and week 8 or 14 (Figure 5C).

**Figure 6:**
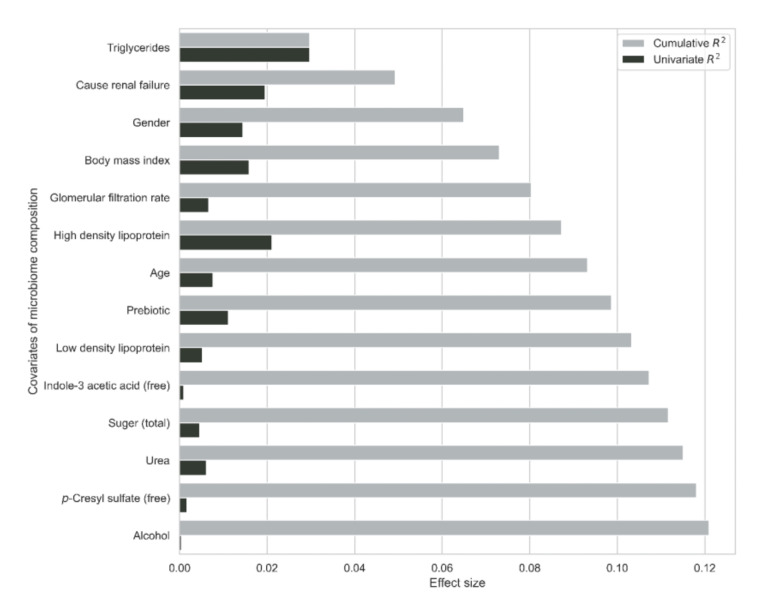
Cumulative effect size of covariates affecting the compositional variation of the gut microbiome selected by the redundancy analysis (grey bars) compared with individual effect sizes assuming independence (black bars).

The redundancy table analysis (RDA) (Figure 6) shows that the following non-redundant factors influenced the gut microbiome’s compositional variation by 12.1%: triglyceride levels (step stepwise dbRDA, R^2^ = 2.97%, *p* < 0.001), the cause of kidney failure (stepwise dbRDA, R^2^ = 1.96%, *p* < 0.001), gender (stepwise dbRDA, R^2^ = 1.56%, *p* < 0.001, BMI (stepwise dbRDA, R^2^ = 0.82%, *p* = 0.002), GFR (stepwise dbRDA, R^2^ = 0.73%, *p* = 0.002), age (stepwise dbRDA, R^2^ = 0.60%, *p* = 0.002), the prebiotic intervention (stepwise dbRDA, R^2^ = 0.55%, *p* = 0.002), high-density lipoprotein (HDL) (stepwise dbRDA, R^2^ = 0.69%, *p* < 0.001), low-density lipoprotein (LDL) (stepwise dbRDA, R^2^ = 0.46%, *p* = 0.008), free IAA (stepwise dbRDA, R^2^ = 0.39%, *p* = 0.016), total sugar intake (stepwise dbRDA, R^2^ = 0.45%, *p* = 0.008), urea (stepwise dbRDA, R^2^ = 0.34%, *p* = 0.019), free *p*CS (stepwise dbRDA, R^2^ = 0.30%, *p* = 0.036), and finally alcohol intake (stepwise dbRDA, R^2^ = 0.29%, *p* = 0.049).

From Table 6, it can be seen that many genera were positively and negatively correlated with the various uremic toxins and other outcomes. *Bifidobacterium* correlated negatively with urea, while *Gemmiger* correlated negatively with creatinine. *Blautia, Clostridium_XlVa, Acetanaerobacterium* correlated positively with TG, while *Prevotella* correlated positively with HDL. *Bulleidia* correlated negatively with total IxS, while *Ruminococcus2* correlated positively. Weight was positively correlated to *Dialister* and *Butyricicoccus,* while BMI was negatively correlated to *Howardella*. *Faecalibacterium* correlated negatively with total *p*CS, while correlating positively with *Methanobrevibacter*, *Desulfovibrio* and *Peptococcus*. Free *p*CS correlated positively with *Escherichia/Shigella.*

## 4. Discussion

In this intervention study, the supplementation of the prebiotic β-glucan resulted in a decrease in especially the free concentrations of the colon-derived uremic toxic levels IxS, pCS and pCG, without a change in kidney function over the 14-week study period. There was a trend to shift from a non-Prevotella to a Prevotella enterotype (going from 43% of subjects having the Prevotella enterotype at baseline to 33% at week 14 in the control group, as opposed to 38% going to 57% in the intervention group, Figure 4), and as there were no changes in dietary intake, these changes may be ascribed to the prebiotic intervention. The RDA showed that the prebiotic significantly affected the gut microbiome.

Previous studies show varying results of prebiotics on kidney function [42]. The effect of prebiotics on kidney function is small [22], in agreement with a recent meta-analysis which showed a reduction in urea, but not creatinine [43]. These changes were not influenced by the dose of fiber, the intervention time or dialysis [43]; the reduction in urea may be due to the fiber intake promoting the growth of colonic bacteria to incorporate nitrogen, resulting in an increased fecal excretion. However, in the present study, no significant differences in the relative abundances of urease-producing genera were observed between groups. In addition, urea may have remained stable owing to the participants’ low protein intake.

Patients with CKD have a high risk of cardiovascular mortality [5]. Dyslipidemia is one of the many traditional risk factors contributing to the development of CVD in the CKD population [44]. In addition, elevated uremic toxins levels have been associated with cardiovascular damage [45]. Dyslipidemia in the later stages of CKD is characterized by increasing total cholesterol and LDL as GFR decreases [46]. In the current study, there was a significant change in LDL cholesterol after 8 weeks, but not at 14 weeks. This reduction may be due to the effect of the ß-glucan prebiotic, since there were no significant changes in dietary intake over time. The ß-glucan prebiotic has been shown to reduce LDL cholesterol in ‘at risk’ CVD patients [23], which is similar to the present findings. Potential mechanisms through which gut microbiota may affect circulating lipid levels could be through the gel-forming property of ß-glucan reducing cholesterol and bile salt absorption, the action of bile salt hydrolase (BSH) [23], as well as through the action of SCFAs [47].

There was a significant reduction in especially the free plasma levels of the colon- derived uremic toxin levels IxS, *p*CS and *p*CG, which might be beneficial since free levels of specific uremic toxins are associated with overall mortality and CVD risk [48]. Additionally, free *p*CS and free IxS were also significantly associated with the gut microbiome according to the RDA analysis. The effect of prebiotics on uremic toxin levels in CKD patients varies; some studies show changes in *p*CS but not IxS. *p*CS and *p*CG are the main conjugates of *p*-cresol, which is generated as an end product of tyrosine metabolism and contributes to uremic toxicity [49]. The reduction in *p*CG is a positive and novel finding and may be linked to the prebiotic fiber intake in the intervention group. Advanced CKD has been linked to increased glucuronidation of *p*-cresol and to CVD and mortality [50]. If fiber is sufficient, gut bacteria use less protein as an energy source, resulting in lower uremic toxin production [48]. Studies have shown significant reductions in *p*CS [51] and IxS [24] with prebiotic use in hemodialysis patients, while synbiotics showed significant changes in *p*CS in predialysis patients [52] and hemodialysis patients [53], with one study showing a reduction in IxS in hemodialysis patients [54]. Wu et al. [55] reported only on a reduction of *p*CS in their meta-analysis of fiber supplementation effects on uremic toxins, while in a very recent meta-analysis, Yang et al. [43] showed that fiber supplementation reduced *p*CS and IxS significantly. They found that the reduction in *p*CS was not influenced by dose, dialysis, diabetes or intervention time, while a significant reduction in IxS was found in dialysis patients. Another possible reason for these positive changes that requires further exploration is that SCFA production alters intestinal integrity, thereby preventing entry of uremic toxins into blood circulation [43].

Gryp et al. [16] reported that the bacterial species involved in phenolic compound generation were mostly from the Bacteroidetes and Firmicutes phyla and suggested targeting Bacteroides in interventions. While studies have shown Prevotella to be negatively associated with pCS and IxS [56], it may be that the trend to shift towards the Prevotella enterotype in the intervention group contributed to the reduction in these uremic toxins. Furthermore, *Desulfovibrio*, *Methanobrevibacter*, and *Peptococcus* were positively correlated with pCS. The abundance of *Desulfovibrio* has been associated with a lower GFR [57]. *Faecalibacterium* was negatively correlated with pCS in the current study; it has been found to be lower in CKD patients and has been found to have a positive correlation with GFR [17,58]. *Methanobrevibacter* has been reported to be pro-inflammatory, while *Peptococcus* is considered to be a pathogen [59,60]. The correlations of the uremic toxins were associated with genera that are reflective of CKD dysbiosis and inflammatory conditions.

The total sugar intake, although within recommended guidelines, was found to significantly affect the gut microbiome according to the RDA. Stanford et al. have linked microbiome variation to individual food groups with a high sugar content [56]. A high sugar intake modifies the ratio of *Bacteroidetes* and *Proteobacteria* to have increased pro-inflammatory properties and reduced immune functioning [61]. The baseline dietary intake was quite varied in this current study. This pattern may have also contributed to baseline differences in enterotype prevalence between the groups.

While the α-diversity was not significantly different, there were differences in the ß-diversity. The higher ß-diversity in the control group may be linked to the lower trend in animal protein intake; studies have shown that microbial diversity is lower in diets high in animal protein [62]. Increased dietary fiber has been linked to a reduction in gut inflammation, which in turn is often associated with low bacterial loads and diversity. Furthermore, fiber has been found to increase *Prevotella* genera [63], which may explain some of the trends seen, owing to the addition of the prebiotic fiber. Prebiotic fiber enhances the selective stimulation of indigenous bacteria in the gut that results in many benefits to the host [62]. Though given transitions from one enterotype to another are rare (Vandeputte D et al., unpublished data), a larger cohort would be required to find significant results here. Studies on the gut microbiome in CKD patients show changes in *Bifidobacterium* and *Lactobacillus* with synbiotics and lactulose [52,64]; however, there were no significant changes in the abundances of these genera in this current study. It may be that these abundances were relatively low to start with. CKD has been associated with lower intestinal colonization of *Bifidobacterium* and *Lactobacillus* [16,65]. However, ß-glucan was found to significantly increase *Bacteroides* and moderately increase *Prevotella* in individuals with elevated cholesterol levels without showing bifidogenic properties [66].

In contrast to previous findings, BSS, a proxy for intestinal transit time, does not explain variation of the gut microbiota composition in this cohort [41,67]. There were no changes over time in gastrointestinal symptoms in both groups. The gut microbiome composition was significantly associated with the TG, HDL, BMI, the prebiotic as well as other factors. The finding of the increased TG and BMI are similar to a study by Viera-Silva et al. [20]. Obesity (35.5%), highly prevalent in participants, has been linked with the progression of CKD [68]. Obesity is associated with an alteration in the gut microbiome [69]. In the current study, the TG and weight were positively associated with various organisms. Zeng et al. [70] identified gut microbiota markers for the classification of obesity-related metabolic abnormalities and over 20 genera associated with multiple clinical factors such as weight, waist circumference and lipid abnormalities. TG show associations with gut microbiome biomarkers that may explain some of the changes in the gut microbiome.

Gut dysbiosis in CKD is closely linked to chronic inflammation [57]. CRP was the only measure of inflammation in the current study; it was high in a majority of participants at baseline but did not change over time. The Bacteroides 2 enterotype, which was particularly prevalent in the control group (18.8% of subjects at baseline) of the current study, may be contributing to inflammation. It has been found to be lower in statin-treated obese individuals; the mechanistic action for this may be in line with the microbiota-inflammation hypothesis [20]. The action of the ß-glucan fiber may be similar to the lipid-lowering effect of statins and has a positive role to play in the change in the composition of the gut microbiome, at least in CKD patients. Statins have been found to positively correlate with secondary gut-derived bile acids that contribute to the magnitude of LDL-lowering [71]. This mechanism of ß-glucan fiber may also be linked to the action of bile salts; the concentration of bile salts seems to affect the proliferation of bacteria [72,73]. In addition, studies suggest that ß-glucan increases BSH activity, which in turn increase cholesterol excretion and bacteria, such as Bifidobacterium, *Bacteroides* and *Lactobaccillus* [74]. There may therefore be different ways that the ß-glucan affects the gut microbiome through the action of bile salts. The exact mechanisms need to be further elucidated.

### 4.1. Strengths of the Study

This single-blind RCT is the first study to examine the effect of the ß-glucan prebiotic on the gut microbiome, uremic toxins and kidney function in parallel in a cohort of participants with CKD not on dialysis using 16S rRNA sequencing methods. It is also the first study to examine enterotypes in this study population as well as the redundancy analysis (RDA), which examined the factors contributing to the gut microbiome variation. Dietary confounders were controlled by using a dietary run-in period, adapted dietary assessment methods and dietary adherence score sheets. Most nutrition status assessment factors remained unchanged during the study, which minimized confounding.

### 4.2. Limitations of the Study

The study limitations include a large number of participant dropouts from pre-randomization to the end of the study, although every effort was made to contact participants for follow-ups. However, this is reflective of a real-time clinic scenario. The duration of the study may have been too short to see an effect on kidney function and cardiovascular outcomes. Although changes were seen in the gut microbiome, a larger number of participants are probably needed to detect significant shifts from non-Prevotella to Prevotella, as well as shifts in the relative abundances in gut microbiota. The gut microbiome in the two groups would be very similar at baseline due to CKD dysbiosis, and one would need larger sample sizes to detect large variations in these groups compared with healthy individuals, where there are distinct differences in the gut microbiome. Dietary intake assessment also has limitations, although the same methodology was applied throughout the study to ensure that the diet intake was adequately assessed and monitored. The lack of a placebo supplement was also a limitation, since patients could not be blinded. However, this did not affect their adherence to the diet.

### 4.3. Recommendations

A study with a larger sample size and of longer duration would be needed to detect significant changes, especially for studies with a focus on changes within the gut microbiome where inter-individual variability is substantial. Post hoc analysis is recommended for future investigation. Other markers of inflammation should be investigated such as interleukin-1ß, interleukin-6, interleukin-10, tumor necrosis factor, and possibly calprotectin in the stool. The findings of the study suggest that the ß-glucan prebiotic fiber supported by simplified CKD dietary advice favorably affects the gut microbiome. Although this study showed benefits with the ß-glucan prebiotic use, more RCTs are needed before generalized recommendations are made. Owing to the high prevalence of obesity found in the group, other measures should also be sought to address these outcomes, such as weight management, behavior modification and physical activity interventions. SCFAs and bile salt levels should be assessed in future studies to investigate the mechanisms involved in cholesterol-lowering and altered gut microbiota composition. Discount vouchers for healthy, natural foods should be sought for this population. The development of a placebo similar in appearance to the prebiotic with minimal nutritional content or effect on the gut microbiome should be considered. Foods containing natural ß-glucans may be encouraged, this can be found in barley, oats, rye and wheat. Although the results of the study cannot be extrapolated to include these foods, they are high in fibre and could be considered as part of a natural, healthy diet.

## 5. Conclusions

In conclusion, the current study found that the supplementation of ß-glucan fiber resulted in reduced plasma levels of the free fraction of colon-derived uremic toxins, as well as a favorable change in the composition of the gut microbiome. We therefore reject the hypothesis that kidney function would change with the prebiotic and accept the hypothesis that uremic toxins and the gut microbiome improved with the prebiotic intervention. The uremic toxins correlated with bacteria that were characteristic of gut dysbiosis. The RDA showed that the prebiotic significantly affected the gut microbiome. Chronic systemic inflammation may be a contributing factor related to the gut dysbiosis in CKD. An increase in uremic toxin levels can contribute to alterations in gut microbiota, resulting in gut dysbiosis, which seems to be exacerbated by metabolic syndrome factors such as obesity and hypertension, which were highly prevalent in the current study population. The mechanism for the changes in this current study may be linked to the lipid-lowering action of the ß-glucan fiber through the action of bile salts and/or attenuation of inflammation, although the only measure of inflammation (CRP) did not show any significant changes.

## Figures and Tables

**Table 1:** Baseline characteristics of participants at randomization.

		Total (*N* = 59)	Intervention Group (*N* = 30)	Control Group (*N* = 29)	*p* Value
**Age (years) (mean)**		41.0 ± 11.6	40.6 ± 11.4	41.3 ± 12.0	0.082
**Socio-demographics**		***N*** **(%)**	***N*** **(%)**	***N*** **(%)**	
**Sex**	Male	25 (42.4)	11 (44)	14(56)	0.367
	Female	34 (57.6)	19 (55.6)	15 (44.1)	
**Income (/month)**	$0−$126	24 (40.7)	10 (41.6)	14 (58.3)	0.332
	$127–$316	16 (27.1)	7 (43.8)	9 (56.2)	
	$317–$633	13 (22.0)	8 (61.5)	5 (38.4)	
	$634–$949	4 (6.8)	3 (75.0)	1 (25.0)	
	>$949	2 (3.4)	2 (100.0)	0	
**Employment**	Employed	29 (49.2)	17 (58.6)	12 (41.3)	0.240
	Unemployed	30 (50.8)	13 (43.3)	17 (56.6)	
**Clinical data**		***N* (%)**	***N* (%)**	***N* (%)**	
**Cause of kidney failure**	Polycystic kidneys	3 (5.1)	1 (33.3)	2 (66.6)	
	Hypertension	29 (49.2)	13 (44.8)	16 (55.2)	0.456
	Glomerular disease	13 (22.0)	9 (69.2)	4 (30.7)	
	Other	14 (23.7)	7 (50.0)	7 (50.0)	
**GFR categories (mL/min/1.73 m^2^)**	30–59 Stage 3	19 (32.2)	9 (47.3)	10 (52.6)	0.867
	15–29 Stage 4	16 (27.1)	9 (56.2)	7 (43.8)	
	<15 Stage 5	24 (40.7)	12 (50.0)	12 (50.0)	
		**Median Interquartile range (IQR)**	**Median (IQR)**	**Median (IQR)**	
**Blood pressure**	**Systolic (mmHg)**	142.0 (128.3, 168,3)	145.0 (134.0, 170.0)	140.0 (136.0, 147.0)	0.549
	**Diastolic (mmHg)**	80.0 (72.0, 92.8)	82.0 (72.0, 93.0)	80.0 (78.0, 91.0)	0.765
**Anthropometry**		**Mean ± standard deviation (SD)**	**Mean ± SD**	**Mean ± SD**	
**Weight (kg)**		75.7 ± 20.7	73.5 ± 18.5	78 ± 22.9	0.461
**BMI (kg/m^2^)**		28.3 ± 6.6	27.4 ± 6.2	29.2 ± 6.99	0.300
**Waist circumference (cm)**		89.6 ± 15.0	86.9 ± 13.1	92.4 ± 16.5	0.161
**MUAC (cm)**		30.2 ± 5.1	29.8 ± 5.2	30.7 ± 5.1	0.527
		***N* (%)**	***N* (%)**	***N* (%)**	
**BMI categories**	Underweight	3 (5.0)	2 (6.7)	1 (3.4)	0.791
	Normal weight	16 (27.1)	10 (62.5)	6 (37.5)	
	Overweight	19 (32.2)	8 (42.1)	11 (57.9)	
	Obese	21 (35.5)	10 (47.6)	11 (52.3)	

Abbreviations: GFR: glomerular filtration rate, IQR: interquartile range, SD: standard deviation, BMI: body mass index, MUAC: mid-upper arm circumference.

**Table 2:** Baseline biochemistry and uremic toxin levels of participants at randomization.

	Total (*N* = 59)	Intervention Group (*N* = 30)	Control Group (*N* = 29)	*p* Value
**Biochemistry**	**Median (IQR)**	**Median (IQR)**	**Median (IQR)**	
**Urea (mmol/L)**	14.2 (9.1, 28.6)	14.4 (11.5, 21.0)	14.1 (10.9, 28.1),	0.952
**Creatinine (mmol/L)**	232.0 (175.0, 461.0)	230.0 (208.0, 53.0)	308.0 (187.0, 33.0)	0.921
**GFR (mL/min.1.73) m^2^**	20.0 (11.0, 35.0)	21.0 (13.0, 27.0)	19.0 (11.0, 35.0)	0.976
**Potassium (mmol/L)**	5.0 ± 0.7	4.9 ± 0.8	5.0 ± 0.7	0.509
**Phosphate** (**mmol/L)**	1.16 (1.0, 1.5)	1.2 (1.1, 1.4)	1.1 (1.0, 1.5)	0.844
**Cholesterol (mmol/L)**	4.7 ± 1.1	4.7 ± 1.1	4.8 ± 1.2	0.958
**LDL (mmol/L)**	2.6 ± 1.0	2.6 ± 0.8	2.6 ± 1.1	0.859
**HDL (mmol/L)**	1.1 (0.9, 1.3)	1.2 (1.1, 1.4)	1.0 (0.9, 1.1)	**0.032**
**Triglycerides (mmol/L)**	1.6 (1.2, 2.4)	1.4 (1.2, 1.9)	1.9 (1.4, 2.6)	0.079
**CRP (mg/L)**	4.0 (2.0, 8.0)	5.0 (2.0, 8.0)	3.0 (2.0, 5.0)	0.760
**Uremic toxins**	**Median (IQR)**	**Median (IQR)**	**Median (IQR)**	
**Total IxS (mg/L)**	3.96 (1.55, 10.27)	3.44 (2.01, 4.93)	5.28 (2.84, 8.32)	0.533
**Free IxS (mg/L)**	0.10 (0.03, 0.26)	0.06 (0.06, 0.14)	0.13 (0.64, 0.22)	0.724
**Total *p*CS (mg/L)**	5.69 (2.86, 10.37)	5.08 (4.20, 7.12)	5.81 (4.09, 7.24)	0.840
**Free *p*CS (mg/L)**	0.14 (0.06, 0.30)	0.14 (0.09, 0.28)	0.14 (0.67, 0.19)	0.391
**Total *p*CG (mg/L)**	0.10 (0.03, 0.26)	0.11 (0.03, 0.21)	0.10 (0.04, 0.17)	0.920
**Free *p*CG (mg/L)**	0.09 (0.02, 0.20)	0.10 (0.03, 0.18)	0.09 (0.03, 0.15)	0.933
**Total IAA (mg/L)**	0.80 (0.49, 1.33)	0.66 (0.55, 1.12)	0.92 (0.70, 1.06)	0.662
**Free IAA (mg/L)**	0.12 (0.09, 0.25)	0.11 (0.10, 0.24)	0.14 (0.11, 0.23)	0.906
**Dietary intake**	**Median (IQR)** **Mean ± SD**	**Median (IQR)** **Mean ± SD**	**Median (IQR)** **Mean ± SD**	
**Energy (kcal)**	5710 (4480.0, 6982.0)	5685.6 (5149.9, 6662.1)	5955.52 (4528.0, 6704.8)	0.773
**Protein (g)**	51.9 ± 20.5	54.3 ± 17.0	49.5 ± 23.7	0.085
**Plant protein (g)**	16.6 (14.0, 21.1)	16.7 (15.1, 19.5)	15.6 (14.5,20.7)	0.544
**Animal protein (g)**	32.5 ± 14.5	34.5 ± 12.0	30.3 ± 16.6	0.127
**Total sugar (g)**	61.5 ± 23	60.3 ± 21.5	62.6 ± 24.7	0.298
**Fiber (g)**	17.6 (14.1, 21.3)	17.6 (16.9, 19.9)	16.2 (14.1, 20.5)	0.448
**Fat intake (g)**	50 (35.1, 60.8)	47.7 (40.5, 53.7)	50.3 (38.4, 55.4)	0.785
**Saturated fat (g)**	13.05 (9.6,18.8)	13.1 (11.7, 17.0)	13.1 (9.4, 15.4)	0.371
**Trans fat (g)**	0.3 (0.1,0.6)	0.3 (0.2, 0.5)	0.3 (0.2, 0.6)	0.844
**Potassium (mg)**	1923.0 (1553.5, 2405.4)	2048.9 (1751.9, 2338.6)	1774.5 (1564.4, 2267.7)	0.396
**Phosphate (mg)**	735.6 (523.0, 939.7)	767.8 (638.6, 865.3)	581.3 (521.4, 848.4)	0.102
**Sodium (mg)**	1829.23 (1290.4, 2584.8)	1999.3 (1666.6, 2435.5)	1803.8 (1186.5, 2090.0)	0.907

Abbreviations: GFR: glomerular filtration rate, LDL: low-density lipoprotein, HDL: high-density lipoprotein, CRP: C-reactive protein, IxS: Indoxyl sulfate, *p*CS: *p*-cresyl sulfate, *p*CG: *p*-cresyl glucuronide, IAA: indole-3-acetic acid. Bold if *p* < 0.005.

**Table 3:** Generalized estimation equation models for change in kidney function over time in the intervention over the control group (proportions).

	Model 1 Outcome: Urea			Model 2 Outcome: Creatinine			Model 3 Outcome: eGFR		
Parameter	Exp (b)	95% CI	*p*	Exp (b)	95% CI	*p*	Exp (b)	95% CI *	*p*
[Intervention group]	0.93	0.66–1.30	0.669	0.92	0.63–0.35	0.625	0.97	0.66–1.43	0.869
[week 8]	1.05	0.92–1.19	0.479	1.02	0.94–1.10	0.613	1.02	0.93–1.12	0.710
[week 14]	1.01	0.88–1.16	0.850	1.01	0.89–1.14	0.891	1.02	0.93–1.12	0.636
[Intervention group] * [week 8]	1.14	0.98–1.33	0.092	1.12	0.97–1.28	0.118	0.95	0.84–1.06	0.244
[Intervention group] * [week 14]	1.06	0.90–1.25	0.514	1.03	0.87–1.22	0.718	0.93	0.81–1.05	0.340

Exp: exponential; b = estimated model coefficient; *p* = *p*-value (Wald χ^2^ test); eGFR: estimated glomerular filtration rate; * Intervention by time treatment effect.

**Table 4:** Generalized estimation equation models for change in lipid values over time in the intervention over the control group (proportions).

	Model 4: Outcome: Total Cholesterol			Model 5 Outcome: LDL Cholesterol			Model 6 Outcome: HDL			Model 7 Outcome: TG		
Parameter	Exp (b)	95% CI	*p*	Exp (b)	95% CI	*p*	Exp (b)	95% CI	*p*	Exp (b)	95% CI	*p*
[Intervention group]	0.98	0.88–1.12	0.997	1.01	0.84–1.24	0.856	1.25	1.02–1.54	**0.031**	0.69	0.50–0.95	0.694
[week 8]	1.02	0.96–1.08	0.476	1.07	0.97–1.17	0.170	1.07	1.02–1.13	**0.010**	0.86	0.75–1.00	0.057
[week 14]	0.98	0.91–1.05	0.594	1.00	0.88–1.14	0.971	1.01	0.91–1.11	0.866	0.90	0.74–1.10	0.307
[Intervention group] * [week 8]	0.93	0.86–1.01	0.571	0.88	0.76–0.99	**0.037**	0.97	0.90–1.05	0.486	1.03	0.84–1.26	0.777
[Intervention group] * [week 14]	1.03	0.93–1.15	0.105	1.02	0.86–1.22	0.776	1.03	0.91–1.15	0.661	1.02	0.81–1283	0.858

Exp: exponential; b = estimated model coefficient; *p* = *p*-value (Wald χ^2^ test); LDL: low density lipoprotein cholesterol; HDL: high density lipoprotein cholesterol; TG: triglycerides, Bold if *p* < 0.05; * Intervention by time treatment effect.

**Table 5:** Generalized expected equations model for changes in plasma levels of uremic toxins over time (mg/L) in the intervention over the control group (proportions).

	Model 1: Outcome: Total IxS			Model 2: Outcome: Free IxS			Model 3: Outcome: Total *p*CS			Model 4 Outcome: Free *p*CS			Model 5 Outcome: Total *p*CG			Model 6 Outcome: Free *p*CG			Model 7 Outcome: Total IAA			Model 8 Outcome: Free IAA		
Parameter	Exp (b)	95% CI	*p*	Exp (b)	95% CI	*p*	Exp (b)	95% CI	*p*	Exp (b)	95% CI	*p*	Exp (b)	95% CI	*p*	Exp (b)	95% CI	*p*	Exp (b)	95% CI	*p*	Exp (b)	95% CI	*p*
[Intervention group]	0.88	0.47–1.62	0.674	1.78	0.65–0.09	0.264	1.16	0.76–1.78	0.479	1.61	0.77–3.40	0.208	3.40	0.86–13.5	0.081	0.34	0.84–1.44	0.087	1.06	0.65–1.74	0.798	1.72	0.77–0.86	0.185
[week 8]	1.08	0.95–1.24	0.211	1.14	0.93–1.40	0.211	0.87	0.83–1.20	0.968	1.06	0.82–1.38	0.630	0.77	0.35–1.87	0.508	0.35	0.12–0.98	**0.045**	1.13	1.00–1.28	0.050	1.13	0.96–1.33	0.148
[week 14]	1.09	0.96–1.21	0.227	1.06	0.84–1.33	0.591	1.00	0.68–1.11	0.258	0.83	0.61–1.12	0.277	1.14	0.77–1.69	0.530	0.16	0.09–0.29	**<0.001**	1.04	0.86–1.26	0.655	1.03	0.84–1.27	0.749
[Intervention group] * [week 8]	0.97	0.71–1.31	0.863	0.46	0.28–0.76	**0.003**	1.09	0.85–1.36	0.952	0.96	0.72–1.28	0.772	0.51	0.15–1.75	0.286	0.45	0.12–1.69	0.241	1.08	0.86–1.35	0.515	0.83	0.50–1.36	0.449
[Intervention group] * [week 14]	0.93	0.68–1.26	0.622	0.35	0.21–0.60	**<0.001**	0.99	0.74–1.32	0.547	0.48	0.29–0.82	**0.006**	0.14	0.08–0.28	**<0.001**	0.13	0.06–0.27	**<0.001**	1.03	0.81–1.31	0.797	0.56	0.31–1.00	0.051

Exp: exponential; b: estimated model coefficient; *p* = *p*-value (Wald χ^2^ test); IxS: Indoxyl sulfate, *p*CS: *p*-cresyl sulfate, *p*CG: *p*-cresyl glucuronide, IAA: indole-3-acetic acid; Bold if *p* < 0.05; * Intervention by time treatment effect.

**Table 6:** Correlations of genera to biochemistry and uremic toxins.

Variable	Genus	* Correlation Value
Urea	*Bifidobacterium*	−0.266
Creatinine	*Gemmiger*	−0.177
LDL cholesterol	*Mogibacterium*	−0.218
HDL cholesterol	*Prevotella*	0.202
TG	*Blautia*	0.197
	*Clostridium_XlVa*	0.205
	*Acetanaerobacterium*	0.180
Weight	*Dialister*	0.236
	*Butyricicoccus*	0.234
BMI	*Howardella*	−0.182
Total IxS	*Ruminococcus2*	0.211
	*Bulleidia*	−0.235
Total *p*CS	*Faecalibacterium*	−0.216
	*Methanobrevibacter*	0.288
	*Desulfovibrio*	0.183
	*Peptococcus*	0.259
Free *p*CS	*Escherichia/Shigella*	0.222
Total IAA	*Mogibacterium*	0.330
	*Howardella*	0.301

Abbreviations: TG: triglycerides, LDL: low-density lipoprotein, HDL: high-density lipoprotein, IxS: Indoxyl sulfate, *p*CS: *p*-cresyl sulfate, *p*CG *p*-cresyl glucuronide, IAA: indole-3-acetic acid * significant correlations.

## Data Availability

Data is confidential but will be made available upon reasonable requests.

## Supplementary Materials

The following are available online at https://www.mdpi.com/article/10.3390/nu14040805/s1, Table S1: Generalized expected equations model for biochemical changes over time in the intervention over the control group (proportions), Table S2: Generalized expected equations model for anthropometrical changes over time in the intervention over the control group (means), Table S3: Generalized expected equations model for dietary intake changes over time in the intervention over the control group (proportions), Table S4: Dietary scores for the intervention and control group over time, Table S5: Relative abundances of genera between groups (ALDEx2). Figures S1–S3: Changes in uremic toxins, Figure S4: Consort 10 cohort mapped onto the FGFP background to determine the enterotype for each sample.

## References

[1] Bikbov B., Purcell C.A., Levey A.S., Smith M., Abdoli A., Abebe M., Adebayo O.M., Afarideh M., Agarwal S.K., Agudelo-Botero M. (2020). Global, regional, and national burden of chronic kidney disease, 1990–2017: A systematic analysis for the Global Burden of Disease Study 2017. Lancet.

[2] Hill N.R., Fatoba S.T., Oke J.L., Hirst J.A., O’Callaghan C.A., Lasserson D.S., Hobbs F.D.R. (2016). Global Prevalence of Chronic Kidney Disease—A Systematic Review and Meta-Analysis. PLoS ONE.

[3] Perico N., Remuzzi G. (2014). Chronic kidney disease in sub-Saharan Africa: A public health priority. Lancet Glob. Health.

[4] Arogundade F.A., Omotoso B.A., Adelakun A., Bamikefa T., Ezeugonwa R., Omosule B., Sanusi A.A., Balogun R.A. (2020). Burden of end-stage renal disease in sub-Saharan Africa. Clin. Nephrol..

[5] Thompson S., James M., Wiebe N., Hemmelgarn B., Manns B., Klarenbach S., Tonelli M. (2015). Cause of Death in Patients with Reduced Kidney Function. J. Am. Soc. Nephrol..

[6] Kim S.M., Song I.H. (2020). The clinical impact of gut microbiota in chronic kidney disease. Korean J. Intern. Med..

[7] Fernandez-Prado R., Esteras R., Perez-Gomez M.V., Gracia-Iguacel C., Gonzalez-Parra E., Sanz A.B., Ortiz A., Sanchez-Niño M.D. (2017). Nutrients Turned into Toxins: Microbiota Modulation of Nutrient Properties in Chronic Kidney Disease. Nutrients.

[8] Chen Y.-Y., Chen D.-Q., Chen L., Liu J.-R., Vaziri N.D., Guo Y., Zhao Y.-Y. (2019). Microbiome–metabolome reveals the contribution of gut–kidney axis on kidney disease. J. Transl. Med..

[9] Ramakrishna B.S. (2013). Role of the Gut Microbiota in Human Nutrition and Metabolism. J. Gastroenterol. Hepatol..

[10] Nallu A., Sharma S., Ramezani A., Muralidharan J., Raj D. (2017). Gut Microbiome in Chronic Kidney Disease: Challenges and Opportunities. Transl. Res..

[11] Vaziri N.D., Zhao Y.Y., Pahl M.V. (2016). Altered Intestinal Microbial Flora and Impaired Epithelial Barrier Structure and Function in CKD: The Nature, Mechanisms, Consequences and Potential Treatment. Nephrol. Dial. Transplant..

[12] Lau W.L., Kalantar-Zadeh K., Vaziri N.D. (2015). The Gut as a Source of Inflammation in Chronic Kidney Disease. Nephron.

[13] Glorieux G., Gryp T., Perna A. (2020). Gut-Derived Metabolites and Their Role in Immune Dysfunction in Chronic Kidney Disease. Toxins.

[14] Ito S., Yoshida M. (2014). Protein-bound Uremic toxins: Protein-Bound Uremic Toxins: New Culprits of Cardiovascular Events in Chronic Kidney Disease Patients. Toxins.

[15] Leong S.C., Sirich T.L. (2016). Indoxyl Sulfate-Review of Toxicity and Therapeutic Strategies. Toxins.

[16] Gryp T., Huys G.R.B., Joossens M., Van Biesen W., Glorieux G., Vaneechoutte M. (2020). Isolation and Quantification of Uremic Toxin Precursor-Generating Gut Bacteria in Chronic Kidney Disease Patients. Int. J. Mol. Sci..

[17] Stanford J., Charlton K., Stefoska-Needham A., Ibrahim R., Lambert L.T. (2020). The Gut Microbiota Profile of Adults with Kidney Disease and Kidney Stones: A Systematic Review of the Literature. BMC. Nephrol..

[18] Vandeputte D., Kathagen G., D’hoe K., Vieira-Silva l., Valles-Colomer M., Sabino J., Wang Y., Tito R.Y., De Commer L., Darzi Y. (2017). Quantitative Microbiome Profiling Links Gut Community Variation to Microbial Load. Nature.

[19] Vieira-Silva S., Sabino J., Valles-Colomer M., Falony G., Kathagen G., Caenepeel C., Cleynen I., van der Merwe S., Vermeire S., Raes J. (2019). Quantitative Microbiome Profiling Disentangles Inflammation- and Bile Duct Obstruction-Associated Microbiota Alterations across PSC/IBD Diagnoses. Nat. Microbiol..

[20] Vieira-Silva S., Falony G., Belda E., Nielsen T., Aron-Wisnewsky J., Chakaroun R., Forslund S.F., Assmann K., Valles-Colomer M., Nguyen T.T.D. (2020). Statin Therapy Is Associated with Lower Prevalence of Gut Microbiota Dysbiosis. Nature.

[21] Valles-Colomer M., Falony G., Darzi Y., Tigchelaar E.F., Wang J., Tito R.Y., Schiweck C., Kurilshikov A., Joossens M., Wijmenga C. (2019). The Neuroactive Potential of the Human Gut Microbiota in Quality of Life and Depression. Nat. Microbiol..

[22] McFarlane C., Ramos C.I., Johnson D.W., Campbell K.L. (2019). Prebiotic, Probiotic, and Synbiotic Supplementation in Chronic Kidney Disease: A Systematic Review And Meta-analysis. J. Ren. Nutr..

[23] Connolly M.L., Tzounis X., Tuohy K.M., Lovegrove J.A. (2016). Hypocholesterolemic and Prebiotic Effects of a Whole-Grain Oat-Based Granola Breakfast Cereal in a Cardio-Metabolic “At Risk” Population. Front. Microbiol..

[24] Valeur J., Puaschitz N.G., Midtvedt T., Berstad A. (2016). Oatmeal Porridge: Impact on Microflora-Associated Characteristics in Healthy Subjects. Br. J. Nutr..

[25] Cosola C., De Angelis M., Rocchetti M.T., Montemurno E., Maranzano V., Dalfino G., Manno M., Zito A., Gesualdo M., Matteo M. (2017). Beta-Glucans Supplementation Associates with Reduction in P-Cresyl Sulfate Levels and Improved Endothelial Vascular Reactivity in Healthy Individuals. PLoS ONE.

[26] Bouhnik Y., Attar A., Joly F., Riottot M., Dyard F., Flourié B. (2004). Lactulose Ingestion Increases Faecal Bifidobacterial Counts: A Randomised Double-Blind Study in Healthy Humans. Eur. J. Clin. Nutr..

[27] NHANES (2007). Anthropometry procedures manual. Natl. Health Nutr. Examinatory Surv..

[28] WHO Body Mass Index. https://www.euro.who.int/en/health-topics/disease-prevention/nutrition/a-healthy-lifestyle/body-mass-index-bmi?source.

[29] Ebrahim Z., Glorieux G., Blaauw R., Moosa M.R.M. (2022). Effect of Simplified Dietary Advice on Nutritional Status and Uremic Toxins in Chronic Kidney Disease Participants. S. Afr. J. Clin. Nutr..

[30] SAFOODS (2017). SAMRC Food Composition Tables for South Africa.

[31] Glorieux G., Vanholder R., Van Biesen W., Pletinck A., Schepers E., Neirynck N., Speeckaert M., De Bacquer D., Verbeke F. (2021). Free p -cresyl Sulfate Shows the Highest Association with Cardiovascular Outcome in Chronic Kidney Disease. Nephrol. Dial. Transplant..

[32] Tito R.Y., Cypers H., Joossens M., Varkas G., Van Praet L., Glorieus E., Van den Bosch F., De Vos M., Raes J., Elewaut D. (2017). Brief Report: Dialister as a Microbial Marker of Disease Activity in Spondyloarthritis. Arthritis Rheumatol..

[33] Tito R.Y., Chaffron S., Caenepeel C., Lima-Mendez G., Wang J., Vieira-Silva S., Falony G., Hildebrand F., Darzi Y., Rymenans L. (2019). Population-Level Analysis of Blastocystis Subtype Prevalence and Variation in the Human Gut Microbiota. Gut.

[34] Callahan B.J., Mcmurdie P.J., Han A.W., Johnson A.J.A., Holmes S.P. (2016). Dada2: High Resolution Sample Inference from Illumina Amplicon Data. Nat. Methods.

[35] Hildebrand F., Tadeo R., Voigt A.Y., Bork P., Raes J. (2014). LotuS: An Efficient and User-Friendly OTU Processing Pipeline. Microbiome.

[36] The Scikit-bio Development Team A Bioinformatics Library for Data Scientists. http://scikit-bio.org.

[37] Pedregosa F., Varoquaux G., Gramfort A., Michel V. (2011). Scikit Learn: Machine Learning in Python. J. Mach. Learn. Res..

[38] Oksanen J., Blanchet F.G., Friendly M., Kindt R., Legendre P., McGlinn D., Minchin P.R., O’Hara R.B., Simpson G.L. (2019). Vegan: Community Ecology Package; Version 2.5-6. https://CRAN.R-project.org/package=vegan.

[39] Fernandes A.D., Reid J.N.S., Macklaim J.M., McMurrough T.A., Edgell D.R., Gloor G.B. (2014). Unifying the Analysis of High-Throughput Sequencing Datasets: Characterizing RNA-Seq, 16S RRNA Gene Sequencing and Selective Growth Experiments by Compositional Data Analysis. Microbiome.

[40] Tackmann J., Matias Rodrigues J.F., von Mering C. (2019). Rapid Inference of Direct Interactions in Large-Scale Ecological Networks from Heterogeneous Microbial Sequencing Data. Cell Syst..

[41] Falony A.G., Joossens M., Wang J., Darzi Y. (2016). Population—Level Analysis of Gut Microbiome Variation. Science.

[42] Koppe L., Fouque D. (2017). Microbiota and Prebiotics Modulation of Uremic Toxin Generation. Panminerva Med..

[43] Yang H.L., Feng P., Xu Y., Hou Y.Y., Ojo O., Wang X.H. (2021). The Role of Dietary Fiber Supplementation in Regulating Uremic Toxins in Patients With Chronic Kidney Disease: A Meta-Analysis of Randomized Controlled Trials. J. Ren. Nutr..

[44] Chen T.K., Knicely D.H., Grams M.E. (2019). Chronic Kidney Disease Diagnosis and Management: A Review. JAMA.

[45] Velasquez M.T., Centron P., Barrows I., Dwivedi R., Raj D.S. (2018). Gut Microbiota and Cardiovascular Uremic Toxicities. Toxins.

[46] Mikolasevic I., Zutelija M., Mavrinac V., Orlic L. (2017). Dyslipidemia in Patients with Chronic Kidney Disease: Etiology and Management. Int. J. Nephrol. Renovasc. Dis..

[47] Vojinovic D., Radjabzadeh D., Kurilshikov A., Amin N., Wijmenga C., Franke L., Ikram M.A., Uitterlinden A.G., Zhernakova A., Fu J. (2019). Relationship between Gut Microbiota and Circulating Metabolites in Population-Based Cohorts. Nat. Commun..

[48] Evenepoel P., Meijers B.K.I., Bammens B.R.M., Verbeke K. (2009). Uremic Toxins Originating from Colonic Microbial Metabolism. Kidney Int..

[49] Koppe L., Alix P.M., Croze M.L., Chambert S., Vanholder R., Glorieux G., Fouque D., Soulage C.O. (2017). P-Cresyl Glucuronide Is a Major Metabolite of p-Cresol in Mouse: In Contrast to p-Cresyl Sulphate, p-Cresyl Glucuronide Fails to Promote Insulin Resistance. Nephrol. Dial. Transplant..

[50] Poesen R., Evenepoel P., de Loor H., Kuypers D., Augustijns P., Meijers B. (2016). Metabolism, Protein Binding, and Renal Clearance of Microbiota-Derived p-Cresol in Patients with CKD. Clin. J. Am. Soc. Nephrol..

[51] Meijers B.K.I., De Preter V., Verbeke K., Vanrenterghem Y., Evenepoel P. (2010). p-Cresyl Sulfate Serum Concentrations in Haemodialysis Patients are Reduced by the Prebiotic Oligofructose-enriched Inulin. Nephrol. Dial. Transplant..

[52] Rossi M., Johnson D.W., Morrison M., Pascoe E.M., Coombes J.S., Forbes J.M., Szeto C.C., McWhinney B.C., Ungerer J.P.J., Campbell K.L. (2016). Synbiotics Easing Renal Failure by Improving Gut Microbiology (SYNERGY): A Randomized Trial. Clin. J. Am. Soc. Nephrol..

[53] Guida B., Germanò R., Trio R., Russo D., Memoli B., Grumetto L., Barbato F., Cataldi M. (2014). Effect of Short-Term Synbiotic Treatment on Plasma p-Cresol Levels in Patients with Chronic Renal Failure: A Randomized Clinical Trial. Nutr. Metab. Cardiovasc. Dis..

[54] Lopes R.D.C.S.O., Theodoro J.M.V., da Silva B.P., Queiroz V.A.V., de Castro Moreira M.E., Mantovani H.C., Hermsdorff H.H., Martino H.S.D. (2019). Synbiotic Meal Decreases Uremic Toxins in Hemodialysis Individuals: A Placebo-Controlled Trial. Food Res. Int..

[55] Wu M., Cai X., Lin J., Zhang X., Scott E.M., Li X. (2019). Association between Fibre Intake and Indoxyl Sulphate/P-Cresyl Sulphate in Patients with Chronic Kidney Disease: Meta-Analysis and Systematic Review of Experimental Studies. Clin. Nutr..

[56] Stanford J., Charlton K., Stefoska-Needham A., Zheng H., Bird L., Borst A., Fuller A., Lambert K. (2020). Associations Among Plant-Based Diet Quality, Uremic Toxins, and Gut Microbiota Profile in Adults Undergoing Hemodialysis Therapy. J. Ren. Nutr..

[57] Li F., Wang M., Wang J., Li R., Zhang Y. (2019). Alterations to the Gut Microbiota and Their Correlation With Inflammatory Factors in Chronic Kidney Disease. Front. Cell. Infect. Microbiol..

[58] Mazidi M., Shekoohi N., Covic A., Mikhailidis D.P. (2020). Role of Anaerostipes Spp. on Renal Function: Insights from a Mendelian Randomization Analysis. Nutrients.

[59] Giri. S., Mangalam A., Faintuch J., Faintuch S. (2019). The Gut Microbiome and Metabolome in Multiple Sclerosis. Microbiome and Metabolome in Diagnosis, Therapy and Other Strategic Applications.

[60] Sufiawati I., Lefaan Y.F.M. (2021). Peptococcus Sp. Associated with Necrotizing Ulcerative Gingivitis in a Child with Leukemia Undergoing Chemotherapy: A Case Report. Int. J. Med. Dent. Case Rep..

[61] Satokari R. (2020). High Intake of Sugar and the Balance between Pro-and Anti-Inflammatory Gut Bacteria. Nutrients.

[62] Simpson H.L., Campbell B.J. (2015). Review Article: Dietary Fibre-Microbiota Interactions. Aliment. Pharmacol. Ther..

[63] Dahl W.J., Rivero Mendoza D., Lambert J.M. (2020). Diet, Nutrients and the Microbiome. Prog. Mol. Biol. Transl. Sci..

[64] Tayebi-Khosroshahi H., Habibzadeh A., Niknafs B., Ghotaslou R., Yeganeh Sefidan F., Ghojazadeh M., Moghaddaszadeh M., Parkhide S. (2016). The Effect of Lactulose Supplementation on Fecal Microflora of Patients with Chronic Kidney Disease: A Randomized Clinical Trial. J. Ren. Inj. Prev..

[65] Sampaio-Maia B., Simões-Silva L., Pestana M., Araujo R., Soares-Silva I.J. (2016). The Role of the Gut Microbiome on Chronic Kidney Disease. Adv. Appl. Microbiol..

[66] Wang Y., Ames N.P., Tun H.M., Tosh S.M., Jones P.J., Khafipour E. (2016). High Molecular Weight Barley β-Glucan Alters Gut Microbiota toward Reduced Cardiovascular Disease Risk. Front. Microbiol..

[67] Vandeputte D., Falony G., Vieira-Silva S., Tito R.Y., Joossens M., Raes J. (2016). Stool Consistency Is Strongly Associated with Gut Microbiota Richness and Composition, Enterotypes and Bacterial Growth Rates. Gut.

[68] Herrington W.G., Smith M., Bankhead C., Matsushita K., Stevens S., Holt T., Hobbs F.D.R., Coresh J., Woodward M. (2017). Body-Mass Index and Risk of Advanced Chronic Kidney Disease: Prospective Analyses from a Primary Care Cohort of 1.4 Million Adults in England. PLoS ONE.

[69] Angelakis E., Armougom F., Million M., Raoult D. (2012). The Relationship between Gut Microbiota and Weight Gain in Humans. Future Microbiol..

[70] Zeng Q., Li D., He Y., Li Y., Yang Z., Zhao X., Liu Y., Wang Y., Sun J., Feng X. (2019). Discrepant Gut Microbiota Markers for the Classification of Obesity-Related Metabolic Abnormalities. Sci. Rep..

[71] Kaddurah-Daouk R., Baillie R.A., Zhu H., Zeng Z.B., Wiest M.M., Nguyen U.T., Wojnoonski K., Watkins S.M., Trupp M., Krauss R.M. (2011). Enteric Microbiome Metabolites Correlate with Response to Simvastatin Treatment. PLoS ONE.

[72] Kakiyama G., Pandak M., Gillevet P.M., Hylemon P.B., Heuman D.M., Daita K., Takei H., Muto A., Nittono H., Ridlon J.M. (2014). Modulation of the Fecal Bile Acid Profile by Gut Microbiota in Cirrhosis. J. Hepatol..

[73] Urdaneta V., Casadesús J. (2017). Interactions between Bacteria and Bile Salts in the Gastrointestinal and Hepatobiliary Tracts. Front. Med..

[74] Joyce S.A., Kamil A., Fleige L., Gahan C.G.M. (2019). The Cholesterol-Lowering Effect of Oats and Oat Beta Glucan: Modes of Action and Potential Role of Bile Acids and the Microbiome. Front. Nutr..

