# Correction: Ebrahim et al. The Effect of ß-Glucan Prebiotic on Kidney Function, Uremic Toxins and Gut Microbiome in Stage 3 to 5 Chronic Kidney Disease (CKD) Predialysis Participants: A Randomized Controlled Trial. *Nutrients* 2022, *14*, 805

**DOI:** 10.3390/nu17193054

**Published:** 2025-09-25

**Authors:** Zarina Ebrahim, Sebastian Proost, Raul Yhossef Tito, Jeroen Raes, Griet Glorieux, Mohammed Rafique Moosa, Renée Blaauw

**Affiliations:** 1 Division of Human Nutrition, Department of Global Health, Stellenbosch University, Cape Town 8000, South Africa; rb@sun.ac.za; 2 Laboratory of Molecular Bacteriology, Department of Microbiology and Immunology, Rega Institute, KU Leuven, 3000 Leuven, Belgium; raulyhossef.titotadeo@kuleuven.be (R.Y.T.); jeroen.raes@kuleuven.be (J.R.); 3 Center for Microbiology, VIB, 3000 Leuven, Belgium; 4 Department of Internal Medicine and Pediatrics, Nephrology Section, Ghent University Hospital, 9000 Ghent, Belgium; griet.glorieux@ugent.be; 5 Department of Medicine, Stellenbosch University, Cape Town 8000, South Africa; rmm@sun.ac.za

After a thorough review of our data and methodology, we identified an issue related to assigning enterotypes to samples from “The Effect of ß-Glucan Prebiotic on Kidney Function, Uremic Toxins and Gut Microbiome in Stage 3 to 5 Chronic Kidney Disease (CKD) Predialysis Participants: A Randomized Controlled Trial” [1].

In the original paper, only two out of four known enterotypes were detected. After review, we found that the enterotyping methodology used in the manuscript contained a minor mismatch, filtering low abundant genera and potential contaminants, between the preprocessing of the cohort and reference data. This issue had a disproportionate impact on the assignment of enterotypes. To address this, we have corrected our preprocessing steps to align the cohort data with standard reference data and reran analyses. As the corrected analyses now show all four enterotypes being present in this cohort, a few corrections to the text are required. Furthermore, this also requires an update of Figure 4 to accurately reflect the findings. We have rectified all issues and provide a corrected version of the text and Figure 4 below, ensuring that both accurately reflect the discussed data and corrected findings.

Fortunately, none of these corrections impact the overall results and conclusions of our study. We do, however, apologize for the oversight and potential consequences this might have had on others’ research.

## 1. Error in Figure

In the original publication [1], there was a mistake in Figure 4 as published. Due to a mismatch between the code and reference data required for detecting the enterotypes, Ruminococcus and Bacteroides 1 samples were detected as Bacteroides 2. The corrected Figure 4 appears below.

**Figure 4:**
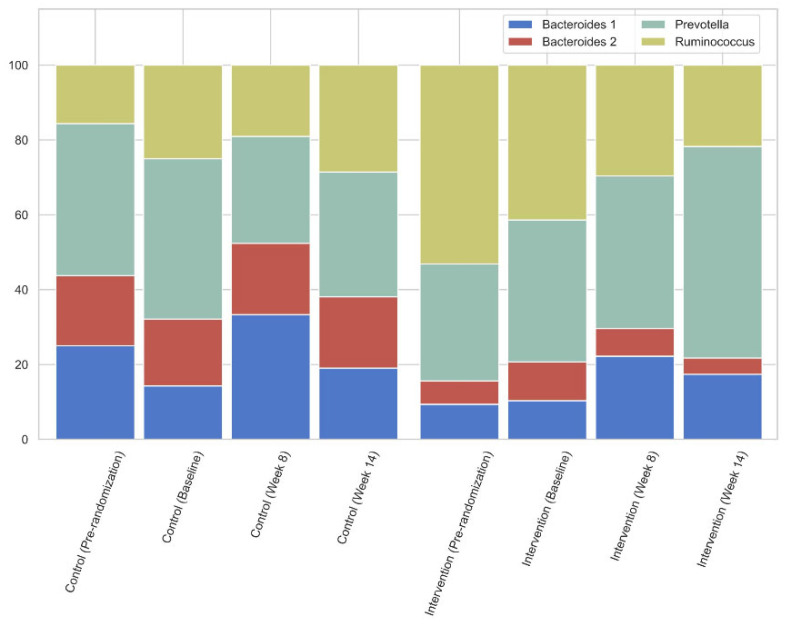
Enterotype percentages of the intervention and control group over time.

## 2. Text Correction

There was an error in the original publication. Due to a mismatch between the code and reference data required for detecting the enterotypes, Ruminococcus and Bacteroides 1 samples were detected as Bacteroides 2. The paragraphs of the text listed below need an update. Note that often a sentence related to Bacteroides 2 was removed. Other changes were highlighted in bold.

A correction has been made to the Abstract (the sentence referring to enterotypes has been removed):

There is growing evidence that gut dysbiosis contributes to the progression of chronic kidney disease (CKD) owing to several mechanisms, including microbiota-derived uremic toxins, diet and immune-mediated factors. The aim of this study was to investigate the effect of a ß-glucan prebiotic on kidney function, uremic toxins and the gut microbiome in stage 3 to 5 CKD participants. Fifty-nine participants were randomized to either the ß-glucan prebiotic intervention group (*n* = 30) or the control group (*n* = 29). The primary outcomes were to assess kidney function (urea, creatinine and glomerular filtration rate), plasma levels of total and free levels of uremic toxins (*p*-cresyl sulfate (*p*CS), indoxyl-sulfate (IxS), *p*-cresyl glucuronide (*p*CG) and indoxyl 3-acetic acid (IAA) and gut microbiota using 16S rRNA sequencing at baseline, week 8 and week 14. The intervention group (age 40.6 ± 11.4 y) and the control group (age 41.3 ± 12.0 y) did not differ in age or any other socio-demographic variables at baseline. There were no significant changes in kidney function over 14 weeks. There was a significant reduction in uremic toxin levels at different time points, in free IxS at 8 weeks (*p* = 0.003) and 14 weeks (*p* < 0.001), free *p*CS (*p* = 0.006) at 14 weeks and total and free *p*CG (*p* < 0.001, *p* < 0.001, respectively) and at 14 weeks. There were no differences in relative abundances of genera between groups. The redundancy analysis showed a few factors significantly affected the gut microbiome: these included triglyceride levels (*p* < 0.001), body mass index (*p* = 0.002), high- density lipoprotein (*p* < 0.001) and the prebiotic intervention (*p* = 0.002). The ß-glucan prebiotic significantly altered uremic toxin levels of intestinal origin and favorably affected the gut microbiome.

A correction has been made to 3. Results, 3.6. Gut Microbiome, Paragraph 2–3 (one sentence has been omitted):

Gut microbiota were characterized by a high relative abundance of *Prevotella* and *Bacteroides,* as shown in Figure 3. This was further confirmed by enterotyping using the Flemish Gut Flora Project as a background (Supplemental Figure S4), where a large subset of participants were assigned to the Prevotella enterotype [41]. There were no significant differences in relative abundances between groups using ALDEx2 (Supplementary data—Table S5).

When comparing the intervention and the control group, as shown in Figure 4, there was a slight downward trend in Prevotella prevalence while increasing in the treatment group.

A correction has been made to 4. Discussion, Paragraph 1 (one sentence has been omitted):

In this intervention study, the supplementation of the prebiotic β-glucan resulted in a decrease in especially the free concentrations of the colon-derived uremic toxic levels IxS, pCS and pCG, without a change in kidney function over the 14-week study period. There was a trend to shift from a non-Prevotella to a Prevotella enterotype (going from 43% of subjects having the Prevotella enterotype at baseline to 33% at week 14 in the control group, as opposed to 38% going to 57% in the intervention group, Figure 4), and as there were no changes in dietary intake, these changes may be ascribed to the prebiotic intervention. **T**he RDA showed that the prebiotic significantly affected the gut microbiome.

A correction has been made to 4. Discussion, Paragraph 5–7 (one sentence has been omitted):

Gryp et al. [16] reported that the bacterial species involved in phenolic compound generation were mostly from the Bacteroidetes and Firmicutes phyla and suggested targeting Bacteroides in interventions. While studies have shown Prevotella to be negatively associated with pCS and IxS [56], it may be that the trend to shift towards the Prevotella enterotype in the intervention group contributed to the reduction in these uremic toxins. Furthermore, *Desulfovibrio*, *Methanobrevibacter*, and *Peptococcus* were positively correlated with pCS. The abundance of *Desulfovibrio* has been associated with a lower GFR [57]. *Faecalibacterium* was negatively correlated with pCS in the current study; it has been found to be lower in CKD patients and has been found to have a positive correlation with GFR [17,58]. *Methanobrevibacter* has been reported to be pro-inflammatory, while *Peptococcus* is considered to be a pathogen [59,60]. The correlations of the uremic toxins were associated with genera that are reflective of CKD dysbiosis and inflammatory conditions.

The total sugar intake, although within recommended guidelines, was found to significantly affect the gut microbiome according to the RDA. Stanford et al. have linked microbiome variation to individual food groups with a high sugar content [56]. A high sugar intake modifies the ratio of *Bacteroidetes* and *Proteobacteria* to have increased pro-inflammatory properties and reduced immune functioning [61]. The baseline dietary intake was quite varied in this current study. This pattern may have also contributed to baseline differences in enterotype prevalence between the groups.

While the α-diversity was not significantly different, there were differences in the ß-diversity. The higher ß-diversity in the control group may be linked to the lower trend in animal protein intake; studies have shown that microbial diversity is lower in diets high in animal protein [62]. Increased dietary fiber has been linked to a reduction in gut inflammation, which in turn is often associated with low bacterial loads and diversity. Furthermore, fiber has been found to increase *Prevotella* genera [63], which may explain some of the trends seen, owing to the addition of the prebiotic fiber. Prebiotic fiber enhances the selective stimulation of indigenous bacteria in the gut that results in many benefits to the host [62]. Though given transitions from one enterotype to another are rare (Vandeputte D et al., unpublished data), a larger cohort would be required to find significant results here. Studies on the gut microbiome in CKD patients show changes in *Bifidobacterium* and *Lactobacillus* with synbiotics and lactulose [52,64]; however, there were no significant changes in the abundances of these genera in this current study. It may be that these abundances were relatively low to start with. CKD has been associated with lower intestinal colonization of *Bifidobacterium* and *Lactobacillus* [16,65]. However, ß-glucan was found to significantly increase *Bacteroides* and moderately increase *Prevotella* in individuals with elevated cholesterol levels without showing bifidogenic properties [66].

A correction has been made to 4. Discussion, Paragraph 9:

Gut dysbiosis in CKD is closely linked to chronic inflammation [57]. CRP was the only measure of inflammation in the current study; it was high in a majority of participants at baseline but did not change over time. The Bacteroides 2 enterotype, which was particularly prevalent in the control group (18.8% of subjects at baseline) of the current study, may be contributing to inflammation. It has been found to be lower in statin-treated obese individuals; the mechanistic action for this may be in line with the microbiota-inflammation hypothesis [20]. The action of the ß-glucan fiber may be similar to the lipid-lowering effect of statins and has a positive role to play in the change in the composition of the gut microbiome, at least in CKD patients. Statins have been found to positively correlate with secondary gut-derived bile acids that contribute to the magnitude of LDL-lowering [71]. This mechanism of ß-glucan fiber may also be linked to the action of bile salts; the concentration of bile salts seems to affect the proliferation of bacteria [72,73]. In addition, studies suggest that ß-glucan increases BSH activity, which in turn increase cholesterol excretion and bacteria, such as Bifidobacterium, *Bacteroides* and *Lactobaccillus* [74]. There may therefore be different ways that the ß-glucan affects the gut microbiome through the action of bile salts. The exact mechanisms need to be further elucidated.

A correction has been made to 4. Discussion, 4.1. Strengths of the Study, Paragraph 1 (a sentence referring to high Bacteroides 2 prevalence has been removed):

This single-blind RCT is the first study to examine the effect of the ß-glucan prebiotic on the gut microbiome, uremic toxins and kidney function in parallel in a cohort of participants with CKD not on dialysis using 16S rRNA sequencing methods. It is also the first study to examine enterotypes in this study population as well as the redundancy analysis (RDA), which examined the factors contributing to the gut microbiome variation. Dietary confounders were controlled by using a dietary run-in period, adapted dietary assessment methods and dietary adherence score sheets. Most nutrition status assessment factors remained unchanged during the study, which minimized confounding.

A correction has been made to 4. Discussion, 4.2. Limitations of the Study, Paragraph 1:

The study limitations include a large number of participant dropouts from pre-randomization to the end of the study, although every effort was made to contact participants for follow-ups. However, this is reflective of a real-time clinic scenario. The duration of the study may have been too short to see an effect on kidney function and cardiovascular outcomes. Although changes were seen in the gut microbiome, a larger number of participants are probably needed to detect significant shifts from non-Prevotella to Prevotella, as well as shifts in the relative abundances in gut microbiota. The gut microbiome in the two groups would be very similar at baseline due to CKD dysbiosis, and one would need larger sample sizes to detect large variations in these groups compared with healthy individuals, where there are distinct differences in the gut microbiome. Dietary intake assessment also has limitations, although the same methodology was applied throughout the study to ensure that the diet intake was adequately assessed and monitored. The lack of a placebo supplement was also a limitation, since patients could not be blinded. However, this did not affect their adherence to the diet.

A correction has been made to 5. Conclusions, Paragraph 1 (a subsentence has been removed):

In conclusion, the current study found that the supplementation of ß-glucan fiber resulted in reduced plasma levels of the free fraction of colon-derived uremic toxins, as well as a favorable change in the composition of the gut microbiome. We therefore reject the hypothesis that kidney function would change with the prebiotic and accept the hypothesis that uremic toxins and the gut microbiome improved with the prebiotic intervention. The uremic toxins correlated with bacteria that were characteristic of gut dysbiosis. The RDA showed that the prebiotic significantly affected the gut microbiome. Chronic systemic inflammation may be a contributing factor related to the gut dysbiosis in CKD. An increase in uremic toxin levels can contribute to alterations in gut microbiota, resulting in gut dysbiosis, which seems to be exacerbated by metabolic syndrome factors such as obesity and hypertension, which were highly prevalent in the current study population. The mechanism for the changes in this current study may be linked to the lipid-lowering action of the ß-glucan fiber through the action of bile salts and/or attenuation of inflammation, although the only measure of inflammation (CRP) did not show any significant changes.

The authors state that the scientific conclusions are unaffected. This correction was approved by the Academic Editor. The original publication has also been updated.
