# Supplementary material for: The Effect of ß-Glucan Prebiotic on Kidney Function, Uremic Toxins and Gut Microbiome in Stage 3 to 5 Chronic Kidney Disease (CKD) Predialysis Participants: A Randomized Controlled Trial
Source: Nutrients. 2022 Feb 14;14(4):805. doi: 10.3390/nu14040805 (PMC8880761; doi:10.3390/nu14040805)
Supplement: Supplementary file 1 [file nutrients-14-00805-s001.zip › nutrients-1485163 - supplementary.pdf]

Supplementary data

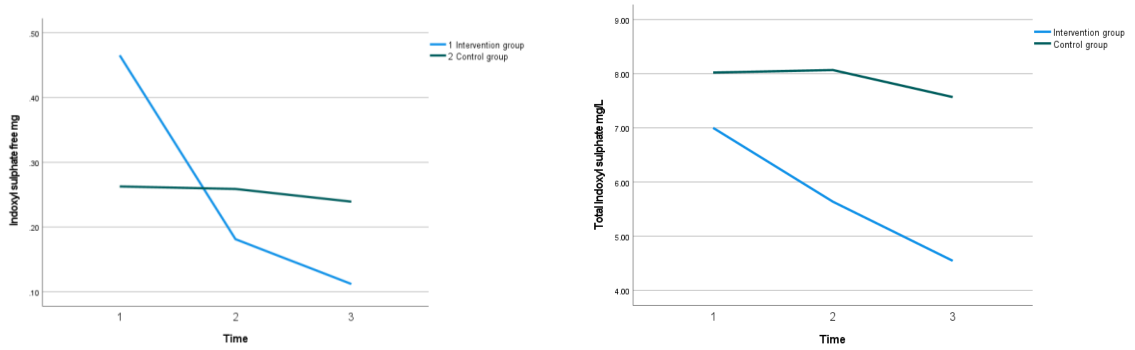

Supplementary Figure S1. Changes in the Intervention and control group in IxS total and free over time.

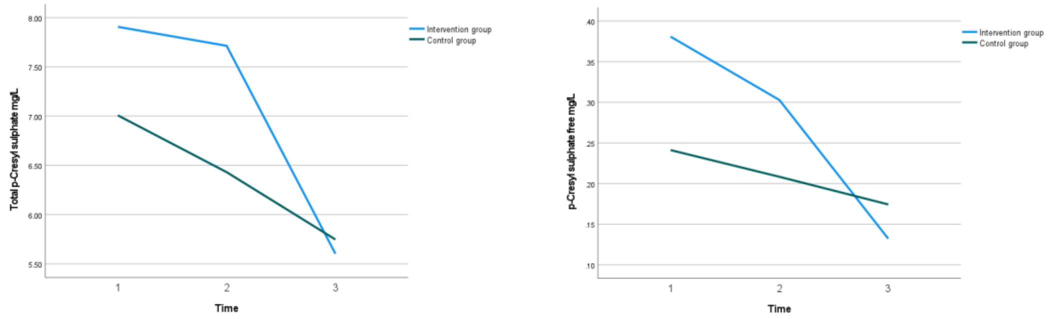

Supplementary Figure S2. Changes in the Intervention and control group in *p*CS total and free over time.

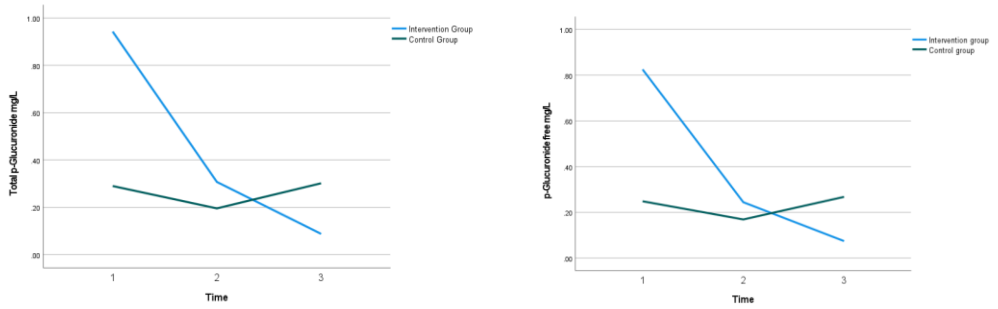

Supplementary Figure S3. Mean changes in the Intervention and control group in *p*CG total and free over time.

Supplementary Table S1. Generalized expected equations model for biochemical changes over time in the intervention over the control group (proportions).

	Model 8: Outcome: CRP mg/L			Model 9: Outcome: Potassium mmol/L			Model 10 Outcome: Phosphate mmol/L		
Parameter	Exp(b)	95% CI	*P*	Exp(b)	95% CI	*P*	Exp(b)	95% CI	*P*
[Intervention group]	0.91	0.34-2.41	0.845	0.99	0.92-1.07	0.796	0.99	0.83-1.14	0.714
[week 8]	0.84	0.46-0.51	0.576	0.97	0.92-1.02	0.169	1.03	0.94-1.12	0.575
[week 14]	0.84	0.51-1.38	0.491	1.01	0.95-1.06	0.789	1.03	0.96-1.11	0.379
[Intervention group] *[week 8]	0.96	0.49-1.88	0.912	1.01	0.99-1.01	0.293	1.10	0.96-1.26	0.185
[Intervention group] *[week 14]	2.21	0.60-8.16	0.232	1.00	0.99-1.02	0.593	0.97	0.87-1.09	0.622

Exp: exponential; b = estimated model coefficient; *P* = *P*-value (Wald χ^2 test); CRP: C-reactive protein

Supplementary Table S2. Generalized expected equations model for anthropometrical changes over time in the intervention over the control group (means).

	Model 1: Outcome: Weight (kg)			Model 2: Outcome: BMI			Model 3: Outcome: Waist circumference (cm)			Model 4 Outcome: MUAC (cm)		
Parameter	Exp(b)	95% CI	*P*	Exp(b)	95% CI	*P*	Exp(b)	95% CI	*P*	Exp(b)	95% CI	*P*
[Intervention group]	4.50	-5.95-14.96	0.398	1.78	-1.51-5.11	0.288	5.05	-1.98-13.0	0.150	0.85	-1.73-3.43	0.518
[week 8]	-0.65	-1.34-0.05	0.070	-0.25	-0.50-0.10	0.057	-0.85	-1.74-0.31	0.059	-0.20	-0.58-0.17	0.291
[week 14]	-0.84	-2.05-0.39	0.181	-0.33	-0.78-0.11	0.147	-0.75	-1.75-0.25	0.142	-0.25	-0.78-0.28	0.362
[Intervention group] *[week 8]	0.88	-1.07-1.25	0.882	0.02	-0.48-0.49	0.985	-0.46	-1.78-0.86	0.492	-0.06	-0.68-0.56	0.840
[Intervention group] *[week 14]	0.43	-1.35-2.21	0.633	0.15	-0.56-0.87	0.678	-0.96	-2.70-0.80	0.286	-0.45	-1.32-0.35	0.252

Exp: exponential; b = estimated model coefficient; *P* = *P*-value (Wald χ^2 test); BMI: body mass index

Supplementary Table S3. Generalized expected equations model for dietary intake changes over time in the intervention over the control group (proportions).

	Model 1: Outcome: Energy (kcal)			Model 2: Outcome: Protein (g)			Model 3: Outcome: Total fat (g)			Model 4: Outcome: Saturated fat (g)			Model 5 Outcome: Diet fibre (g)			Model 6 Outcome: Potassium (mg)			Model 7 Outcome: Phosphate (mg)			Model 8 Outcome: Sodium (mg)		
Parameter	Exp(b)	95% CI	*P*	Exp(b)	95% CI	*P*	Exp(b)	95% CI	*P*	Exp(b)	95% CI	*P*	Exp(b)	95% CI	*P*	Exp(b)	95% CI	*P*	Exp(b)	95% CI	*P*	Exp(b)	95% CI	*P*
[Intervention group]	0.96	0.80-1.17	0.693	1.10	0.89-1.3	0.375	0.98	0.77-1.25	0.858	1.08	0.83-1.40	0.556	1.05	0.88-1.24	0.603	1.05	0.88-1.26	0.541	1.12	0.91-1.34	0.282	0.72	0.71-1.28	0.787
[week 8]	0.94	0.83-1.06	0.312	0.93	0.80-1.10	0.407	0.85	0.72-1.00	**0.035**	0.88	0.74-1.04	0.135	1.10	0.98-1.22	0.093	1.00	0.90-1.11	0.945	0.96	0.83-1.11	0.592	0.70	0.70-1.11	0.297
[week 14]	0.90	0.76-1.08	0.266	0.95	0.78-1.15	0.601	0.84	0.64-1.10	0.203	0.83	0.63-1.10	0.206	1.10	0.95-1.24	0.219	1.02	0.88-1.20	0.768	0.97	0.80-1.12	0.822	0.62	0.62-1.16	0.322
[Intervention group] * [week 8]	1.00	0.84-1.19	0.990	0.96	0.80-1.17	0.695	1.08	0.87-1.34	0.455	0.78	0.80-1.21	0.833	0.91	0.78-1.07	0.294	0.96	0.83-1.11	0.962	0.95	0.80-1.13	0.555	0.73	0.73-1.44	0.879
[Intervention group] * [week 14]	1.08	0.85-1.36	0.530	0.98	0.78-1.23	0.903	1.15	0.82-1.60	0.403	0.80	0.80-1.57	0.501	0.93	0.77-1.12	0.458	0.93	0.76--1.13	0.934	0.96	0.76-1.22	0.761	0.80	0.81-1.86	0.341

Exp: exponential; b = estimated model coefficient; *P* = *P*-value (Wald χ^2 test)

Supplementary Table S4. Dietary adherence scores for the intervention and control group over time.

Group	Baseline (%)	4 weeks (%)	Week 8 (%)	Week 14 (%)
Intervention	89.4	93.3	94.6	94.2
Control	85.6	92.3	91.0	91.7

Supplementary Table S5. Relative abundances.

Genera	Adj p value (Welch)	Adj p value (Wilcoxin)	Relative abundance overall	Relative abundance within Control	Relative abundance within Intervention	Difference between groups	Difference within groups	Effect size	Overlap between groups
Prevotella	0.27	0.36	3.23	2.29	3.70	1.03	7.96	0.12	0.44
Faecalibacterium	0.63	0.68	6.36	6.36	6.35	0.11	2.12	0.04	0.48
Roseburia	0.14	0.04	5.54	5.92	5.12	-0.77	2.84	-0.23	0.38
Blautia	0.46	0.93	5.47	5.37	5.52	0.03	1.78	0.02	0.49
Bifidobacterium	0.74	0.93	1.94	1.69	2.21	-0.13	6.51	-0.02	0.49
Catenibacterium	0.51	0.61	1.55	1.70	-1.16	-0.34	8.87	-0.04	0.48
Bacteroides	0.75	0.47	5.18	5.44	4.87	-0.39	3.52	-0.10	0.45
Ruminococcus	0.55	0.72	4.33	4.39	4.28	-0.19	2.51	-0.05	0.47
Ruminococcus 2	0.62	0.75	4.15	4.20	4.11	-0.10	2.00	-0.04	0.48
Dorea	0.20	0.27	3.74	3.85	3.67	-0.32	1.86	-0.14	0.43
Holdemanella	0.85	0.81	3.11	3.12	3.10	-0.19	7.76	-0.03	0.48
Fusicatenibacter	0.91	0.86	3.04	2.93	3.15	0.09	2.55	0.03	0.49
Coprococcus	0.88	0.84	3.48	3.49	3.48	-0.09	2.14	-0.03	0.48
Clostridium _IV	0.56	0.33	3.38	3.63	3.17	-0.31	2.15	-0.12	0.44
Clostridium _XIVa	0.37	0.37	2.63	2.45	2.92	0.39	2.99	0.11	0.44

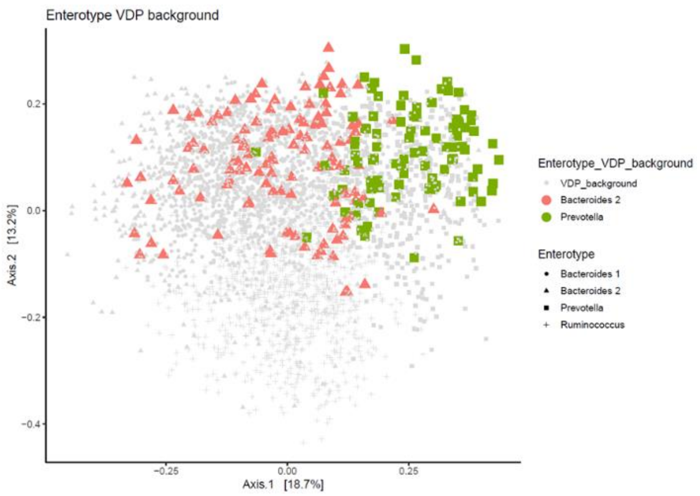

Supplementary Figure S4. Consort 10 cohort mapped onto the FGFP background to determine the enterotype for each sample.
